# GPR40 partial agonists and AgoPAMs: Differentiating effects on glucose and hormonal secretions in the rodent

**DOI:** 10.1371/journal.pone.0186033

**Published:** 2017-10-20

**Authors:** Michele J. Pachanski, Melissa E. Kirkland, Daniel T. Kosinski, Joel Mane, Boonlert Cheewatrakoolpong, Jiyan Xue, Daphne Szeto, Gail Forrest, Corin Miller, Michelle Bunzel, Christopher W. Plummer, Harry R. Chobanian, Michael W. Miller, Sarah Souza, Brande S. Thomas-Fowlkes, Aimie M. Ogawa, Adam B. Weinglass, Jerry Di Salvo, Xiaoyan Li, Yue Feng, Daniel A. Tatosian, Andrew D. Howard, Steven L. Colletti, Maria E. Trujillo

**Affiliations:** 1 In Vivo Pharmacology, Merck & Co., Inc., Kenilworth, New Jersey, United States of America; 2 Translational Imaging Biomarkers, Merck & Co., Inc., Kenilworth, New Jersey, United States of America; 3 Department of Medicinal Chemistry, Merck & Co., Inc., Kenilworth, New Jersey, United States of America; 4 Department of Cardio Metabolic Diseases, Merck & Co., Inc., Kenilworth, New Jersey, United States of America; 5 Pharmacokinetics, Pharmacodynamics and Drug Metabolism, Merck & Co., Inc., Kenilworth, New Jersey, United States of America; Tohoku University, JAPAN

## Abstract

GPR40 agonists are effective antidiabetic agents believed to lower glucose through direct effects on the beta cell to increase glucose stimulated insulin secretion. However, not all GPR40 agonists are the same. Partial agonists lower glucose through direct effects on the pancreas, whereas GPR40 AgoPAMs may incorporate additional therapeutic effects through increases in insulinotrophic incretins secreted by the gut. Here we describe how GPR40 AgoPAMs stimulate both insulin and incretin secretion in vivo over time in diabetic GK rats. We also describe effects of AgoPAMs in vivo to lower glucose and body weight beyond what is seen with partial GPR40 agonists in both the acute and chronic setting. Further comparisons of the glucose lowering profile of AgoPAMs suggest these compounds may possess greater glucose control even in the presence of elevated glucagon secretion, an unexpected feature observed with both acute and chronic treatment with AgoPAMs. Together these studies highlight the complexity of GPR40 pharmacology and the potential additional benefits AgoPAMs may possess above partial agonists for the diabetic patient.

## Introduction

GPR40 AgoPAMs were first described by Luo as a potential therapy to ameliorate two of the major hormonal deficiencies in type 2 diabetes through the stimulation of insulin and GLP-1 secretion [1]. Since this report, several investigations have revealed a complex cellular pharmacology for GPR40 agonists separating these synthetic free fatty acid-like molecules into two classes: 1) the partial agonist such as AMG 837, TAK-875, and MK-8666, and 2) the AgoPAM such as AM-1638 and additional compounds AP1 and AP3 described herein [1–3]. Comparisons between compounds of these classes in vitro have demonstrated differences in receptor binding, the level to which they stimulate receptor activation, and in the case of AgoPAMs the added aspect of incretin secretion [1]. In vivo, the GPR40 AgoPAM- stimulated cell based effects on the beta and/ or enteroendocrine cells result in greater glucose lowering and enhanced incretin secretion that are target-mediated with a single dose in the mouse. These data are intriguing and open the possibility that development of a GPR40 AgoPAM may provide the advantage of combination therapies consisting of efficacy driven by the GPR40 plus that of GLP-1 based therapies (such as DPP4 inhibitors or GLP-1 analogs) in one small molecule oral anti-diabetic therapeutic.

Most of the effects described above comparing GPR40 partial agonists and AgoPAMs have been conducted in vitro. However, some data demonstrating the increased insulin secretory effects and elevation in GLP-1 secretion have been shown acutely in vivo. Specifically, acute treatment with AgoPAMs results in greater increases in insulin and GLP-1 in mice [1,4]. Furthermore, these properties are hypothesized to contribute to greater glucose lowering acutely in vivo with AgoPAMs compared to partial agonists. These acute data of the effects of AgoPAMs in mice were corroborated by, and extended in, diabetic GK rats where AgoPAMs were shown to have enhanced glucose lowering compared to partial agonist; an effect that was durable up to two weeks of treatment [5]. The long term effects of AgoPAM treatment on insulin and GLP-1 secretion are not known.

GPR40 partial agonists are clinically validated glucose lowering agents [6]. GPR40 agonists provide efficacy with minimal risk of hypoglycemia; this property is thought to be due to: 1) the glucose dependent nature of GPR40 partial agonist effects on insulin secretion, and 2) that insulin secretion by the beta cell is thought to be the primary driver of glucose lowering with partial agonist therapy [7,8]. Combination of GPR40 partial agonists with DPP4 inhibitors has never been tested clinically and both therapies are thought to work through increases in glucose dependent insulin secretion via the beta cell. The potential for these therapies to work complimentary is supported by the differential signaling mechanisms where partial agonists work through Gq and DPP4 inhibitors work through increases in GLP-1 resulting in increased Gs signaling pathways in the beta cell [9]. AgoPAMs increase insulin secretion both by direct stimulation at the beta cell and indirectly through GLP-1 secretion, similar to the partial agonist combined with GLP-1 (through DPP4 inhibition or GLP-1 analog therapies).

The opportunity to develop a GPR40 AgoPAM delivers the potential for enhanced efficacy compared to the partial agonist and additional benefits such as increased satiety, weight loss, and CV benefits due to increases in GLP-1 with this therapy. Our explorations into the comparison of hormonal effects of AgoPAMs vs. partial agonists in the diabetic GK rat reveal some intriguing and sometimes unexpected results.

## Research design and methods

### Human, rat, and mouse GPR40 IP1 accumulation assays

Stable cell lines expressing human GPR40 (hGPR40/HEK293), mouse GPR40 (mGPR40/CHO-K1), and rat GPR40 (rGPR40/CHO-K1) were cultured in DMEM media supplemented with 10% FBS, glutamine, non-essential amino acids, and penicillin/streptomycin. hGPR40/HEK293 & mGPR40/CHO-K1 cell media was supplemented with 500 mg/ml G418 (Life Technologies), while rGPR40/CHO-K1 cells were grown in 10 mg/ml blasticidin plus 200 mg/ml hygromycin (Life Technologies). Cell stocks were maintained and grown in a sub-confluent state using standard cell culture procedures. The day before the experiment, the cells were harvested with non-enzymatic cell dissociation buffer and re-suspended in DMEM supplemented with 10% FBS, glutamine, non-essential amino acids, penicillin/streptomycin at 0.15, 0.2 and 0.3 million cells per ml for human, mouse, and rat GPR40, respectively. A sterile Perkin Elmer Culturplate-384 was then seeded with 7,500, 10,000 or 15,000 human, mouse, or rat GPR40 cells in a volume of 50 μl per well. The seeded plates were incubated overnight at 37 C.

On the day of the experiment, the growth media was removed from the assay by gently patting the assay plates on an absorbent sheet and 10 μl of IP1 stimulation buffer (Cis Bio IP-one Tb HTRF kit) supplemented with 50 mM LiCl is added to each well. Test compounds dissolved in DMSO were serially diluted in ½ log increments starting from 2 mM or 0.2 mM and 50 nl of the compound dilution was acoustically added to each well (final starting concentration 10 μM or 1 μM). Plates were then incubated for 60 minutes at room temperature and 10 μl of detection buffer (prepared as described in the Tb kit) is added to each well. The plates are then incubated one additional hour at room temperature. After the final incubation, the plates were read in a Perkin Elmer Envision with a method designed for HTRF assays (320 nm excitation, dual emission 615 and 655 nm). For each assay, a standard curve plate in which IP1 is titrated is also included. All fluorescent readings (using the 655/615 nm ratio) are back calculated to a concentration of IP1 using the IP1 standard curve. The percent activity at each concentration of test compound is determined using 0% activation (basal activity) determined in those wells that contain DMSO alone, while 100% activity is determined in wells that contained a concentration of a partial allosteric agonist know to activate GPR40. The % activity is then plotted versus the concentration of test compound and the dose response curve fitted to a standard 4-parameter non-linear regression model using a custom in-house developed software package (Merck & Co., Inc., MRL, Kenilworth, NJ, USA). Maximal % activity and EC50 are then determined for each test compound.

### Binding assay

The GPR40 binding assay measures affinity at two unique sites on GPR40 that are allosterically coupled [2]. Two small molecule tracers, the partial agonist [3H]L358 and the AgoPAM [3H]25, are used to measure compound affinity at each site as described previously [5]. Because of the allosteric coupling of the two sites and the nature of the assay, addition of a compound will either displace (binds to same site) or augment (binds to allosterically coupled site) radiotracer.

Membranes expressing GPR40 were prepared from CHO cells stably expressing human GPR40. Cell pellets were re-suspended in homogenization buffer (10 mM Tris HCl (KD Medical RGF-3340), 1mM EDTA (Sigma)) plus Protease inhibitor tablets (1 tab per 100 ml buffer; Sigma P8830) and were homogenized in 50 ml aliquots with a motor-driven glass Dounce homogenizer, 15 strokes at 1900 rpm on wet ice, repeating once. Homogenates were centrifuged at 2200 rpm for 10 min at 4°C. Supernatants were carefully removed and pooled. Homogenization and centrifugation were repeated until the debris pellet was translucent to maximize membrane recovery. Supernatants were then centrifuged at 18K rpm for 20 min. at 4°C. Pellets were re-suspended in membrane storage buffer (50 mM Tris HCl, 2.5 mM EDTA, 5 mM MgCl2 Sigma M1028, 100g/L Sucrose Sigma S9378, Protease inhibitor 1μL/ml Sigma P8340) and passed successively through 21g, 23g and 25g needles, aliquoted, flash frozen in liquid nitrogen and stored at -80°C. Protein concentration was determined by Pierce BCA kit.

For the binding assay, WGA Yitrium SPA beads (PE) are diluted to a concentration of 25 mg/ml in binding buffer (50 mM Tris HCl, pH7.4; 10 mM MgCL2; and 2 mM EDTA) to which protease inhibitors (SigmaFast Cocktail) have been added. GPR40 membranes are prepared by gently passing an aliquot of stock membranes through a 25 gauge needle (BD). The homogenized membranes are diluted to a concentration of 20 mg/ml in binding buffer containing 0.1% BSA. Beads and membranes are then combined to a working concentration of 86 μg/ml GPR40 membranes and 4300 μg/ml SPA beads. The beads and membrane mixture is then vigorously mixed for 30 minutes at room temperature prior to addition to the assay. Radioligand tracers, [3H]-L358 (SA 53 Ci/mmol, Merck & Co., Inc., MRL, Kenilworth, NJ, USA) and [3H]25 (SA 69 Ci/mmol, Merck & Co., Inc., MRL, Kenilworth, NJ, USA), are diluted to a concentration of 6.6 nM and 16.7 nM respectively in binding buffer containing 0.5% BSA. Test compounds dissolved in DMSO were serially diluted in ½ log increments starting from 2 mM, and 500 nl of the compound dilution was acoustically added to each well of the assay plate (PE OptiPlate 384). Next, 15 μl of the radioligand and 35 μl of the beads/membranes solution is added to each well. The assay plates are then sealed and incubated for 30 minutes at room temperature on an orbital shaker. The plates are allowed to settle for 16 hours and then counted on a TopCount scintillation counter (PE). For data analysis, percent bound at each concentration of test compound is determined using 100% bound determined in those wells that contain DMSO alone, while 0% bound is determined in wells that contained a concentration of a standard compound known to maximally displace the tracer. The % bound is then plotted versus the concentration of test compound and the dose response curve fitted to a standard 4-parameter non-linear regression model using a custom in-house developed software package. Maximal % bound and IC50 are then determined for each test compound.

5 μg/well membrane together with 200 μg/well of PVT WGA SPA beads (GE #RPNG-0001) were suspended in a white 96 well plate with a final concentration of 50 μl/well in assay buffer (50 mM Tris, 10 mM MgCl2, and 2 mM EDTA). The plate was shaken aggressively for 30 mins before adding 20 μl/well of compound in 0.1% BSA assay buffer and 30 μl/well of radioligand in 0.5% assay buffer yielding a final concentration of 15,000 cpm/well. The plate is briefly mixed and incubated for 3–18 h at room temperature, before it is read on a MicroBeta plate counter (Perkin Elmer, MS, USA).

### Animal use and care

Male Goto Kakizaki (GK) rats received at 8 weeks of age and age matched Wistar Kyoto (WKY) lean controls (Taconic Farms Inc., Germantown, NY) were housed in pairs on a 12 h light cycle (lights on 7 A.M.– 7 P.M.). Rats were allowed access to rodent chow (Rodent Diet #5L0D) and water ad libitum and acclimated for at least 1 week prior to study. RD #5L0D was purchased from LabDiets (Richmond, IN). Compound was formulated into RD #5L0D by Research Diets Inc. (New Brunswick, NJ).

Wild type male C57BL/6N mice (Taconic Farms), aged 12–16 weeks, were housed in groups of 8. Male GPR40-/- mice and male GLP-1R-/- mice originally described in Lan et al [10] were housed in groups of 4–5. All mice were on a 12 h light cycle (lights on 7 A.M.– 7 P.M.). Mice were allowed access to rodent chow (RD #5053, LabDiets, St. Louis, MO) and water ad libitum and acclimated for at least 1 week prior to study. All procedures were approved by the Institutional Animal Care and Use Committee of Merck & Co., Inc., Kenilworth, NJ USA.

### GK rat acute efficacy

Ambient blood glucose was measured via tail snip with a glucometer (OneTouch Ultra, Lifescan Inc., Milpitas, CA). Body weights were then measured, and food was removed approximately 1 hr post lights on. Animals were sorted into treatment groups based on ambient glucose. At approximately 10 AM, glucose was measured and blood for insulin, glucagon, and drug exposure was collected. Immediately following, the rats were dosed PO at a volume of 5 ml/kg with either vehicle (0.5% methylcellulose), TAK-875 at 100 mg/kg, AP1 at 30 mg/kg, or AP3 at 30 mg/kg. TAK-875 is a partial GPR40 agonist [11] and AP1 and AP3 are two structurally distinct AgoPAMs synthesized at Merck & Co., Inc., Kenilworth, NJ USA [5,12]). Each compound tested was dosed at its maximal effective dose determined through a series of dose titrations using this experimental paradigm (data not shown). Effects of treatment on glucose, insulin, and glucagon were measured at 1, 2, 4, and 24 hrs post dose. After the 4 hr blood collection, food was returned overnight and removed the next day at 1 hr post lights on.

### GK rat chronic efficacy

At the start of the study, fed (ambient) blood glucose was measured via glucometer at 1 h post lights on, and animals were selected and sorted into treatment groups based on ambient glucose (n = 10/ GK group, n = 6 WKY control). Blood for baseline incretin measurements was collected in the PM via tail vein. Rats were dosed via oral gavage (PO) at a volume of 5 ml/kg daily at approximately 12:30 PM with either vehicle (0.5% methylcellulose), AP1 at 30 mg/kg, or AP3 at 30 mg/kg. MK-8666, a GPR40 partial agonist synthesized at Merck & Co., Inc., Kenilworth, NJ USA [13], at 30 mg/kg was administered in feed (Research Diets, New Brunswick, NJ) and provided ad lib. All compounds were administered to achieve maximal efficacious exposures determined in acute efficacy studies similar to those acute studies described above. In brief, partial agonists and AgoPAMs were titrated in the acute GK rat experiment described above and the exposure associated with maximal efficacy in glucose lowering was noted. Partial agonists and AgoPAMs were then dosed to maintain those exposures up to 24h post dose. In the chronic experiment MK-8666 was used instead of TAK-875 though the two partial agonists are very similar in their pharmacological properties and again, MK-8666 was dosed to achieve its maximal efficacious exposure in the chronic setting for a proper comparison with the two AgoPAMs. Rats from this group and the WKY controls were administered vehicle PO when the vehicle, AP1, and AP3 groups were treated. Fed blood glucose, food intake, and body weights were determined on days 10, 17, 23, and 28 of study at 2 h post lights on. Immediately after these measures were taken on days 10, 17, 23, and 28, food was removed. After 5h of fasting, animals were dosed with vehicle or compound and allowed to rest for an additional hour. Glucose, GLP-1, GIP, and PYY were measured after 6h of fasting at 1 hr post dose. Fed blood glucose is described by animals having had ad libidum access to feed up till the time of sampling and occurred at 2h post-lights on where rats were nearing the end of their nocturnal feeding cycle. Fasted blood glucose is described by blood glucose sampled after 6h of fasting where food was removed at 2h post lights on. Please note that the fasted sample was taken 1h post oral dosing. On day 10, blood was collected from the tail vein (20 μl) after morning glucose measures and after 6 hour fast from a subset of n = 3 rats per treatment group to determine the fed and fasted exposure of the compounds in circulation. The blood was stored at 4°C in 0.1 M sodium citrate for subsequent analysis of drug exposure.

### Target engagement

To calculate estimated GPR40 target engagement in the rodent studies, intrinsic EC50 values estimated from IP1 accumulation assay experiments, fraction unbound (fu) measurements, and drug concentration (Conc) measurements from collected blood samples were used. Blood concentrations of test compounds were determined by LC-MS/MS following protein precipitation with acetonitrile. Percent target engagement was calculated using the equation:

$$TE\left(\%\right)=\frac{100\%\times Conc\left(nM\right)}{Conc\left(nM\right)+\frac{EC_{50}\left(nM\right)}{f_{u}}}$$

### Isolation of pancreatic islets and the static GDIS assay

Pancreatic islets of Langerhans were isolated from wild type and GPR40^−/−^ mice (littermates) by collagenase digestion and discontinuous Ficoll gradient separation [14]. The islets were cultured overnight in RPMI-1640 medium with 11 mmol/l glucose to facilitate recovery from the isolation process. Insulin secretion was determined by a 1-h static incubation in Krebs-Ringer bicarbonate (KRB) buffer in a 96-well format as previously described [15,16]. Briefly, islets were first preincubated in KRB medium with 2 mmol/l glucose for 30 min and were then transferred to a 96-well plate (three islets/well) and incubated with 200 μl of the KRB medium with 15 mmol/l glucose in the presence or absence of testing compounds for 60 min. The buffer was removed from the wells at the end of the incubation and assayed for insulin levels using the Insulin ELISA kit (ALPCO, Salem, NH) and glucagon level using glucagon ELISA kit (ALPCO, Salem, NH).

### Endogenous glucose production in vivo

To evaluate the effects of the GPR40 partial and AgoPAM compounds on EGP in vivo, a 2H/13C dual tracer approach was used to calculate EGP and its contributing sources. Following the chronic treatments described above, 0.9% NaCl salinated deuterium oxide (D2O, Sigma-Aldrich, St. Louis, MO) was administered to all animals via intraperitoneal (IP) injection at dose volume of 20 ml/kg at 1 hr post lights on. Animals were then placed in clean cages and food was removed. After a 5 hour fast, blood glucose was measured and animals were dosed with 50 mg/kg [U-13C] glucose (Sigma-Aldrich, St. Louis, MO) IP at 5 ml/kg. Blood was collected via tail snip at time points from 60–150 min post treatment to measure blood glucose (via glucometer) as well as endogenous (12C-glucose) and exogenous (13C-glucose) via GCMS. Following the last tail blood collection, rats were anesthetized with isoflurane and cardiac blood was collected into K2EDTA tubes (Becton, Dickinson and Co., Franklin Lakes, NJ).

The serially collected blood samples were analyzed for 13C-glucose enrichment using GC-MS analysis. The % 13C glucose enrichments were then combined with the total glucose readings to calculate the concentration of 13C-glucose, and these values were modeled with a two-compartment model of whole body glucose metabolism to calculate the average EGP for the period from 0–150 minutes [17].

Terminal blood samples were analyzed for relative 2H enrichment in plasma glucose using the monoacetone glucose method [18]. Briefly, samples were extracted with 70% perchloric acid at a ratio of 50 μl perchloric acid per 1 ml plasma and centrifuged at 25,000 rcf for 10 min at 4°C. The supernatant was collected and the pH was adjusted to 6–8 using aqueous KOH. The samples were then lyophilized overnight. To convert plasma glucose to monoacetone glucose (MAG), acetone and concentrated sulfuric acid were added to the lyophilized samples and stirred for 4 hours at room temperature. Water was then added and the pH adjusted to 2.0 with 3M Na2CO3. Samples were stirred moderately for 18 hours at room temperature, the pH was adjusted to 7 with 3M Na2CO3, and the samples were completely dried by speed vacuum concentration. The resulting MAG samples were then extracted in hot ethyl acetate, dried overnight under a stream of N2 gas, and purified using Discovery DSC-18 SPE columns (6 ml, 1 g bed weight). For the SPE protocol, MAG samples were reconstituted in 3 ml water and passed over the columns which were pre-washed with 2 ml methanol. The columns were rinsed twice with 1 ml water and the MAG was eluted with 5% acetonitrile. The acetonitrile eluent was then transferred to a glass scintillation vial and freeze dried. Purified MAG samples were solubilized in 180 μl 90% acetonitrile for 2H NMR analysis which was performed using the lock channel of a 950 MHz Bruker NMR spectrometer (David H Murdock Research Institute, DHMRI Kannapolis, NC). Acquisition parameters were as follows: 90° pulse, D1 = 1 s, 1H broadband decoupling, NS = 3600, acquisition time ~ 1 hr. The 2H NMR spectra of the MAG samples were then analyzed using Matlab (The Mathworks Inc, Natick, MA) to model the NMR peaks with a Lorentzian lineshape, and the results were used to calculate integrals for each of the seven NMR resonances in the MAG spectrum. Peaks 2, 5, and 6s were then used to calculate the relative sources of EGP (i.e. gluconeogenesis from TCA cycle substrates, glucose production from triose phosphates, and glycogenolysis) and these values were combined with the total EGP measurement to calculate absolute fluxes of glucose production.

### ^13^C-labeled glucose tolerance test

On day 0, GK rats were selected and sorted into treatment groups based on ambient glucose measured via glucometer at 1 h post lights on. MK-8666 at 30 mg/kg was administered in feed (Research Diets) and provided ad lib on day 0. On days 1–6, rats were dosed via oral gavage (PO) at a volume of 5 ml/kg daily at approximately 12:30 PM with either vehicle (0.5% methylcellulose) or AP1 at 10 mg/kg. Rats from the MK-8666 group were administered vehicle PO at this time. On day 7, ambient glucose was measured 1 hr post lights on and food was removed. After approximately 5 hrs fasting, glucose was measured at time = - 60 min, followed by PO administration of vehicle or AP1. At time = 0, animals were challenged PO at 10 ml/kg dose volume with [1-13C] glucose prepared in distilled water at 1 g/kg (Sigma-Aldrich, St. Louis, MO). Blood glucose was then measured at t = 20, 40, 60, 120 minutes post challenge. Following the blood glucose measurement at time = 120 min, animals were anesthetized with Isoflurane (Isothesia, Henry Schein, Dublin, OH) and samples of liver and skeletal muscle were collected.

Tissue samples were processed using a methanol/water based extraction procedure as follows. Frozen tissue was weighed and added to tubes containing 80% methanol at a ratio of 100 mg tissue per ml 80% methanol. The samples were homogenized at 4°C using a Polytron homogenizer equipped with a 12mm stainless steel head. Samples were then centrifuged at 25,000 rcf for 10 min at 4°C, the supernatant collected, and the pellet extracted again using an equivalent volume of 80% methanol. The supernatant from both extractions was pooled and the methanol evaporated under nitrogen. The samples were then reconstituted in 180 μl 0.1M sodium phosphate in D_2_O (pH 7.4) for NMR analysis. 13C NMR of tissue extracts was performed on a 600 MHz Bruker NMR spectrometer (DHMRI) using the following parameters: 45° pulse, D1 = 0.2 sec, NS = 3200, 1H broadband decoupling (WALTZ-16), acquisition time 45 min. The following 13C NMR signals were recorded: glucose-6-phosphate C1 (97 ppm), lactate C3 (21 ppm), alanine C3 (17.1 ppm). These NMR signals were integrated and converted to absolute units (μmoles) using 13C NMR spectra from standard solutions of each metabolite of known concentration acquired under identical conditions.

Glycogen is a primary site of glucose disposal in both liver and muscle; however glycogen was not preserved in our tissue extraction protocol. To quantify tissue 13C-glycogen, a separate piece of tissue from each sample was analyzed [19]. Briefly, tissue samples were saponified in hot KOH, and this was followed by glycogen precipitation with NaSO_4_ and ethanol, and then degradation with amyloglucosidase. The resulting glycogen-derived glucose solution was analyzed for total glucose using a glucose oxidase / horseradish peroxidase assay kit and fluorescence-based product detection according to the manufacturers’ instructions (BioVision Glycogen Assay Kit, Milpitas, CA). Fractional 13C enrichment in glucose was performed using GC-MS and this data was combined with the total glucose measurements to yield the tissue concentration of 13C-glycogen.

### Mouse incretin measurements

Wild type and GPR40-/- mice were weighed the day prior to dosing. Food was removed from mice at lights on. 2 hours later mice were dosed via oral gavage (PO) at a volume of 10 ml/kg with vehicle (0.5% methylcellulose), AP1 at 30 mg/kg, or AP3 at 30 mg/kg. At 60 minutes post dose, mice were euthanized via CO_2_ asphyxiation. Blood was collected via cardiac puncture.

### Mouse glucose tolerance test

Wild type C57BL/6 mice and GLP-1R^-/-^ mice were fed a high fat diet (D12492i, Research Diets) for 6 weeks prior to GTT. On the day of study, mice were fasted at 1 h post lights on, and body weights were measured. Following a 6 hr fast, glucose was measured via tail vein at time = - 60 min. Mice were then dosed PO at a volume of 10 ml/kg with vehicle (0.5% methylcellulose), MK-2305 (a GPR40 partial agonist described in Hauge et al [2]) at at 10 mg/kg, or AP1 at 30 mg/kg. Dosages of MK-2305 and AP1 were administered at maximal efficacious doses as determined by acute dose titrations with efficacy determined via reduction in glucose during an IPGTT (data not shown). MK-2305 was used as a representative partial agonist in mice as it has better mouse PK compared to TAK-875 or MK-8666 and therefore a better tool for experimentation. Blood glucose was measured again (time = 0) just prior to an intraperitoneal (IP) dextrose challenge of 1 g/kg at 10 ml/kg dose volume (Dextrose, Hospira Inc., Lake Forrest IL). A subset of vehicle treated mice was injected with 0.9% NaCl saline to serve as negative control. Blood glucose was then measured at 10, 20, 40, 60, and 120 min after challenge. To capture insulin secretion during the IPGTT, blood via tail vein was collected at t = - 60, 0, 10, 20, 40, 60 min post challenge.

The blood glucose excursion profile from t = 0 to t = 120 min was used to integrate a glucose area under the curve (AUC) for each treatment. The plasma insulin excursion profile from t = 0 to t = 60 min was used to integrate an insulin AUC for each treatment. Net AUC was calculated by subtracting the average AUC of the saline group from each groups’ total AUC.

### Measurement of plasma hormones

Blood for insulin, glucagon, GLP-1, GIP, and PYY measurements was collected into K2EDTA Microtainer tubes (BD, Franklin Lakes, NJ) pre-treated with DPP4 inhibitor (Millipore, Billerica, MA) and cOmplete^™^ Protease Inhibitor Cocktail Tablets (Roche Diagnostics, Indianapolis, IN). Tubes were centrifuged for 10 minutes at 5000 RPM, and plasma was aliquotted for analysis. Plasma insulin levels were measured via EIA (Mesoscale Discovery ELISA Kit, Gaithersburg MD). Rat plasma total GLP-1 was measured using Total GLP-1 (ver.2) Assay Kit (Cat#: K150JVC). The measurement of active GLP-1 was carried out in a similar manner as that described for total GLP-1 assay with the exception that MSD Active GLP-1 (ver. 2) assay kit (Cat# K150JWC) was used. Rat plasma glucagon was measured using an in-house glucagon assay developed as a MSD format sandwich immunoassay (Merck & Co., Inc., MRL, Kenilworth, NJ USA). The antibody specificity was cross validated via mass- spec using procedures described in Lee et al [20]. Briefly, MSD Streptavidin Gold plates were coated with 2 μg/ml biotin mouse monoclonal antibody generated to glucagon. Test samples were added to the plates and a separate Sulfo- tagged monoclonal antibody was used to detect the captured glucagon protein (2 μg/ml). The ELISA was carried out in the standard reagents in the MSD format. The plate was read on the SECTOR Imager 6000 Reader. Metabolic hormones GIP (Gastric Inhibitory Polypeptide) and PYY (Peptide YY) were measured in rat plasma using the Rat Metabolic Magnetic Bead Panel (EMD Millipore, Billerica, MA). The assay was performed according to manufacturer protocol.

### Data analysis

All data are presented as mean ± SEM, except where indicated. Comparisons among groups were made using ordinary one-way ANOVA (followed by Dunnett’s or Tukey’s multiple comparison posttests) or unpaired Student's t test (when comparing only two test groups), as appropriate. P < 0.05 was regarded as statistically significant.

## Results

### GPR40 in vitro characterization

**Fig 1:**
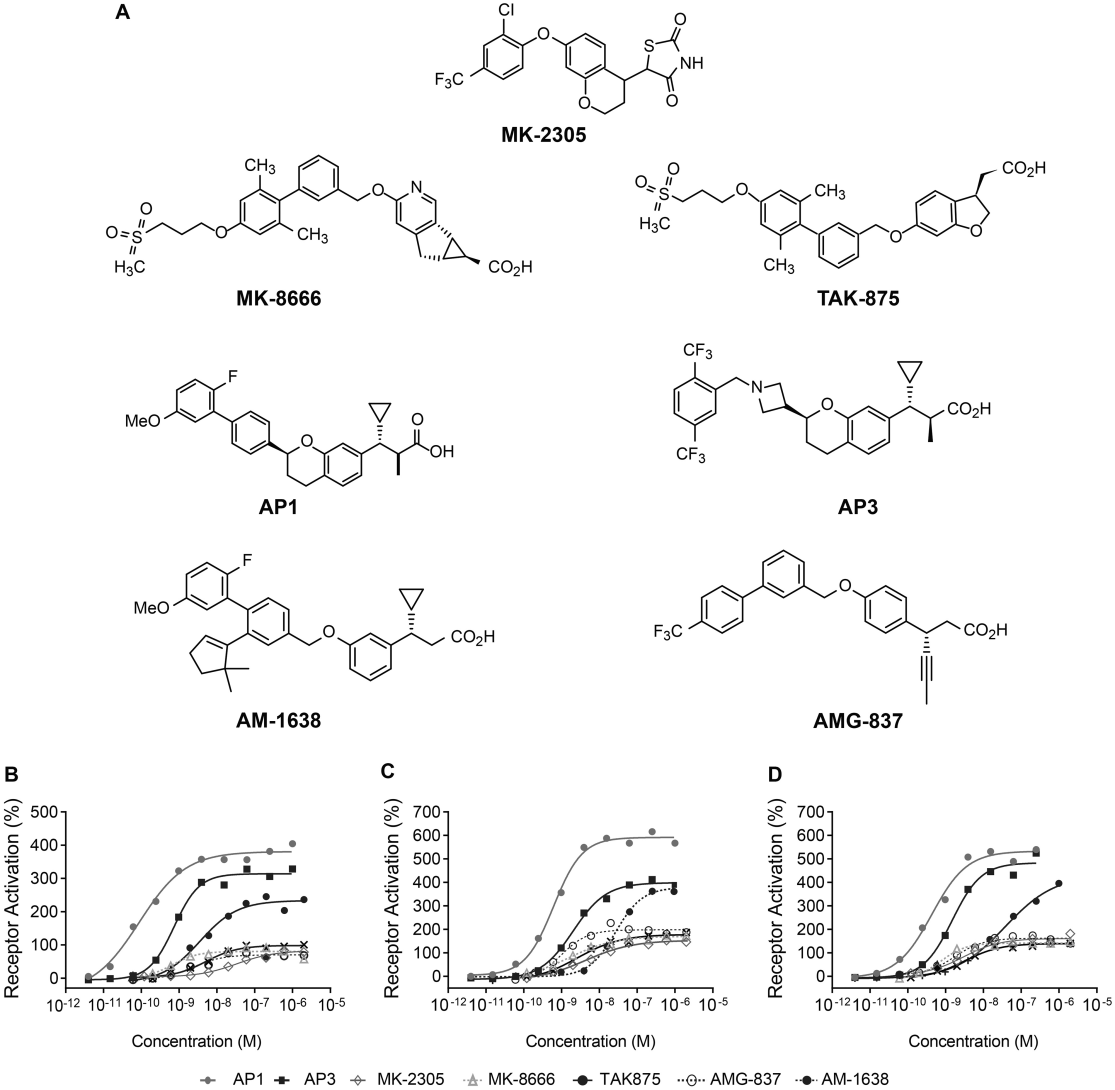
Chemical structures and measurement of IP accumulation in stable cell lines expressing human, rat, or mouse GPR40. (A) Chemical structures of partial agonists MK-8666, MK-2305, TAK-875, AMG-837; and AgoPAMs AM-1638, AP1, and AP3. Dose-response curves for partial agonists (MK-8666, MK-2305, TAK-875, and AMG-837) and AgoPAMs (AM-1638, AP1, and AP3) were generated in human GPR40/CHO-K1 (B), rat GPR40/HEK293 (C), or mouse GPR40/HEK293 (D) cells. Data are expressed as a percentage of the control response of an in-house partial agonist and fitted to a standard 4-parameter non-linear regression model. EC50’s were determined using a custom in-house developed software package. Each compound was profiled multiple times (n = 2–20) with representative graphs shown. The mean parameters of these are shown in Table 1.

The structures of compounds from both GPR40 partial agonist and AgoPAMs compared in vitro and in vivo described herein are displayed in Fig 1A. In vitro, previously characterized GPR40 partial allosteric agonists (AMG-837) and AgoPAMs (AM-1638) show differentiated levels of receptor activation in recombinant cells expressing relatively low levels of human, rat, or mouse GPR40 by measuring the accumulation of inositol monophosphate (IP1), a product of downstream signaling (Table 1)[5,21]. Notably, using these cell reagents, two AgoPAMs, AP1 (Cpd24^2^ in Plummer et al, Merck & Co., Inc., Kenilworth, NJ USA [5]) and AP3 [12] demonstrate comparable efficacy to AM-1638 and differentiate from the partial allosteric agonists AMG-837, TAK-875 and MK-8666 (Fig 1B–1D & Table 1).

**Table 1:** Functional properties of partial agonists and AgoPAMs in human, rat, and mouse GPR40 IP accumulation assays.

Potency [EC_50_, nM] ± SD and Efficacy (% Activation)			
	Human	Rat	Mouse
AP1	0.15 ± 0.05	0.59 ± 0.28	0.51 ± 0.12
	(359%)	(531%)	(532%)
AP3	0.48 ± 0.25	1.3 ± 0.63	1.2 ± 0.72
	(318%)	(405%)	(478%)
MK-2305	24 ± 13	5.2 ± 2.5	2.6 ± 0.8
	(85%)	(155%)	(157%)
MK-8666	0.54 ± 0.22	1.6 ± 0.69	0.89 ± 0.41
	(86%)	(149%)	(143%)
TAK-875	4.0 ± 2.2	7.8 ± 4.0	4.4 ± 2.1
	(102%)	(158%)	(145%)
AMG-837	2.2 ± 0.6	1.5 ± 0.41	1.9 ± 0.76
	(78%)	(173%)	(149%)
AM-1638	3.7 ± 1.9	41 ± 21	36 ± 11
	(200%)	(373%)	(402%)

To assure that AP1 and AP3 bind to human GPR40 in a similar manner to AM-1638, competition binding experiments were performed in membranes from stably transfected CHO-K1 cells. Two different radioligands were used: 1) [^3^H]L358 [2,5], a thiazolidinedione allosteric partial agonist, binding in a similar manner to AMG-837 and TAK-875 [3,21]; and 2) [^3^H]25, a study compound utilized following binding studies demonstrating that GPR40 partial allosteric agonists and AgoPAMs demonstrated identical profiles with [^3^H]AM-1638 and [^3^H]25 ([5], data not shown). Like AM-1638, AP1 and AP3 displace the binding of [^3^H]25, consistent with an AgoPAM binding mode and clearly contrasting with MK-8666, MK-2305, AMG-837, and TAK-875, which displace the binding of [^3^H]L358 (Table 2).

**Table 2:** Summary of [3H]L358 and [3H]AP9 ([3H]25,) binding to WT human GPR40. Each compound was profiled multiple times (n = 2–13). N.A. specifies that a compound augmented the binding of specified radioligand in line with the positive cooperativity between the partial agonist and AgoPAM binding sites.

Binding Results [IC_50_, nM]		
	[^3^H]L-358	[^3^H]AP9
AP1		2.7 ± 0.7
		(101%)
AP3		5.9 ± 5.2
		(101%)
MK-8666	5.6 ± 2.6	
	(88%)	
MK-2305	17 ± 4.7	
	(108%)	
TAK-875	18 ± 11	
	(91%)	
AMG-837	24 ± 15	
	(92%)	
AM-1638		20 ± 8.3
		(101%)

### Gpr40 partial agonist vs. AgoPAM effects on glucose lowering, insulin, and incretins

Previously, GPR40 AgoPAMs have been described to have greater glucose lowering effects compared to partial agonists acutely in mice during a glucose challenge and in hyperglycemic models [1]. To assess this comparison more rigorously over time and to capture the interplay of pancreatic and gut hormones that may be involved in mediating the differential effects of GPR40 partial agonists (MK-8666 and TAK-875) and two structurally distinct AgoPAMs (AP1 and AP3) (Fig 1A), we performed a series of single and multiple dose studies in the diabetic GK rat.

**Fig 2:**
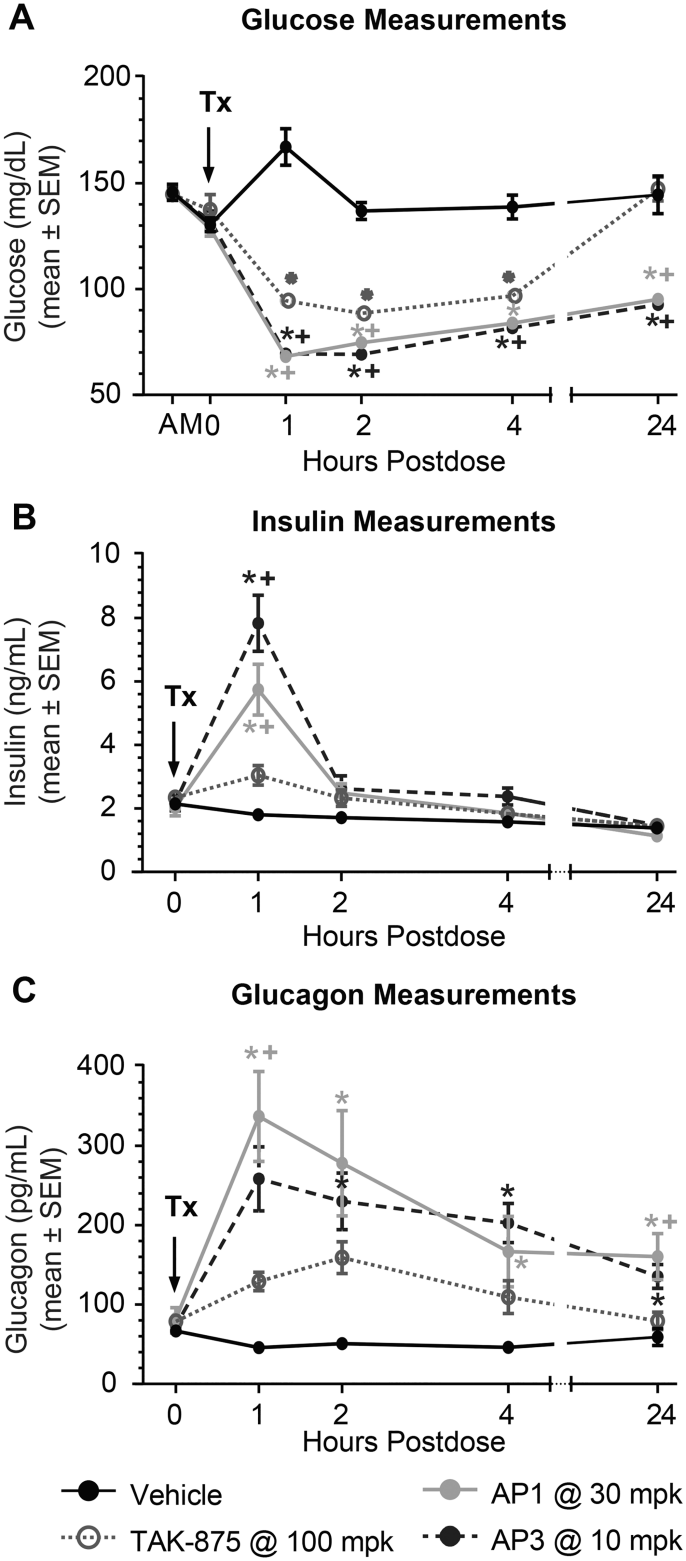
Acute treatment with GPR40 partial agonists and AgoPAMs in GK rats. One hour post-acute treatment with vehicle, TAK-875 at 100 mg/kg, AP1 at 30 mg/kg, or AP3 at 10 mg/kg, blood glucose dropped to its lowest levels (A) at the same time insulin peaked (B) in GK rats. All compounds were dosed at maximal efficacious doses. Acute treatment with the AgoPAMs produced a significant 24 hr increase in glucagon, while the glucagon increase of TAK-875 was not significant and was of a shorter duration (C). Data are mean ± SEM with analysis via ANOVA followed by Tukey’s posttest. * *P < 0*.*05* vs. Vehicle, ^+^ *P < 0*.*05* vs. TAK-875.

To determine the dynamics of glucose, pancreatic hormones, and gut hormones following a single dose, GK rats were treated with either vehicle, TAK-875 at 100 mg/kg (a partial agonist), AP1 at 30 mg/kg, or AP3 at 10 mg/kg (both AgoPAMs). To enable an objective comparison between partial and AgoPAM agonist biological effects, the degree of estimated target engagement can be compared. At these doses, the estimated %TE for these compounds were in the range of 73% - 93% for the early portion of single dose studies (in the 2–6 hr range) for all three compounds, with AP1 having the lowest and TAK-875 and AP3 comparably higher values. Further results on drug exposures and % TE for both acute and chronic studies can be found in S2 and S3 Tables and S1 Fig 1. At 1 hr post dose, for all GPR40 agonist treatments, blood glucose dropped to its lowest levels (Fig 2A) at the same time insulin peaked (Fig 2B) in GK rats. The magnitude of the effect of GPR40 AgoPAMs on both insulin (at the 1 hr time point) and glucose (total AUC) were much larger than that observed with the partial agonist. After the hyperglycemic glucose levels in the GK rat were corrected to euglycemia following administration of partial agonist, insulin was observed to return to baseline and remained at baseline levels for the remainder of the study. Glucose levels remained reduced for AgoPAMs throughout the 24 hr time course compared to vehicle, while the glucose in rats treated with TAK-875 was reduced from the 1 hr time point until the 8 hr time point, after which the reductions in glucose lowering with TAK-875 treatment diminished. The loss of efficacy with TAK-875 treatment at the 24 hr time point was likely due to loss of drug exposure needed to drive the glucose lowering, having calculated target engagement for TAK-875 between 1–6 hr post treatment of at least ~80% TE, and reduced levels at approximately 44% at the 24 hr time point (S1 Table and S2 Fig). In contrast, AP3 and AP1 maintained approximately 80% TE up through 24 hr post dose. Most interestingly, acute treatment with the AgoPAMs produced a significant increase in glucagon for up to 24 hr, while the glucagon increase of the partial agonist was not significant (Fig 2C). Results observed with TAK-875 were nearly identical to those observed with MK-8666 administered at a maximally efficacious dose in a similar but separate series of experiments.

**Fig 3:**
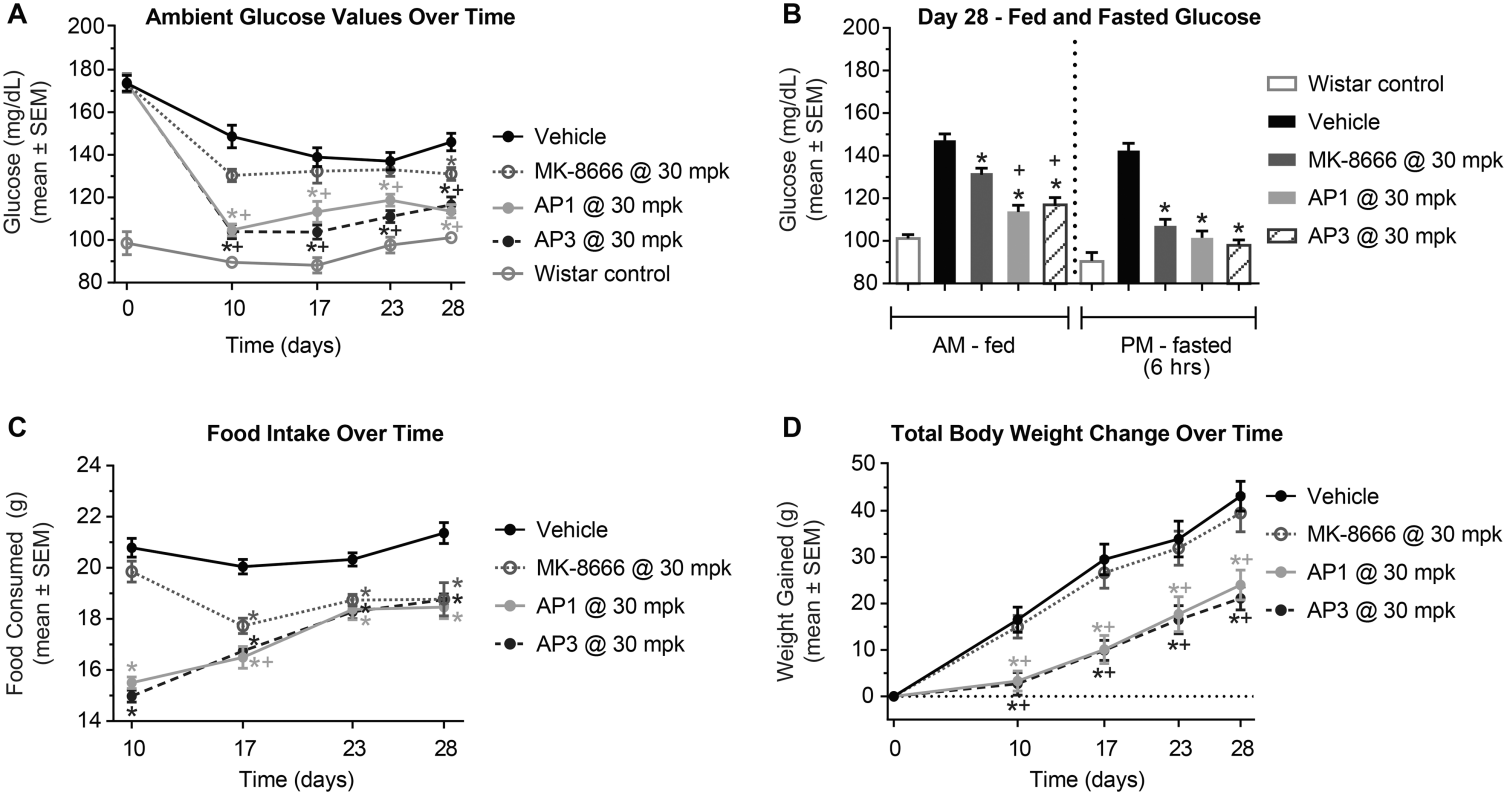
GK rat glucose, body weight, and food intake are affected by 28 days treatment with AgoPAMs or partial agonist. All compounds were dosed at maximal efficacious doses. Fed blood glucose levels were significantly reduced by GPR40 AgoPAMs compared to vehicle controls on days 10 through 28 of treatment, but only on day 28 by the partial agonist (P < 0.05, A). On day 28 of study, fed and fasted blood glucose was significantly reduced with GPR40 partial and AgoPAM treatments compared to vehicle controls (P < 0.05, B). Additionally, AP1 significantly reduced fed blood glucose compared to the partial agonist (P < 0.05, B). Decreased levels of food intake were observed with AgoPAM treatment days 10 through 28 (P < 0.05), while the partial agonist did not reduce food intake until day 17 (P < 0.05, C). The effects of AgoPAMs on food intake were associated with decreases in body weight (P < 0.05, D) whereas MK-8666 had no effect on body weight. Data are mean ± SEM with analysis via ANOVA followed by Tukey’s posttest. * *P < 0*.*05* vs. Vehicle, ^+^ *P < 0*.*05* vs. MK-8666.

To assess this difference between GPR40 partial and AgoPAM agonists on efficacy and determine the durability of these effects, GK rats were treated with vehicle, MK-8666 at 30 mg/kg, AP1 at 30 mg/kg, or AP3 at 30 mg/kg for 28 days. MK-8666 was used instead of TAK-875 for this experiment as we were ost interested in which Merck compound would be most beneficial to patients based on this preclinical work. GPR40 AgoPAMs significantly reduced fed blood glucose levels compared to vehicle controls on day 10 through day 28 of treatment (*P < 0*.*05*, Fig 3A). In contrast, MK-8666, the partial agonist, had a significant effect on day 28 only. Additionally, AP1 significantly reduced fed blood glucose compared to the partial agonist (*P < 0*.*05*, Fig 3B). In the fasted state, blood glucose was reduced by all treatments compared to vehicle controls, but there was no difference between the partial agonist and AgoPAMs. While a small and consistent ~10% decrease in food intake has been observed over time previously with partial agonists, AgoPAM treatments showed a marked reduction in food intake at earlier time points that seemed to level off, similarly to that seen with partial agonists by d17, of treatment (*P < 0*.*05*, Fig 3C). The effects of AgoPAMs on food intake were associated with decreases in body weight (*P < 0*.*05*, Fig 3D) whereas MK-8666 had no effect on body weight.

**Fig 4:**
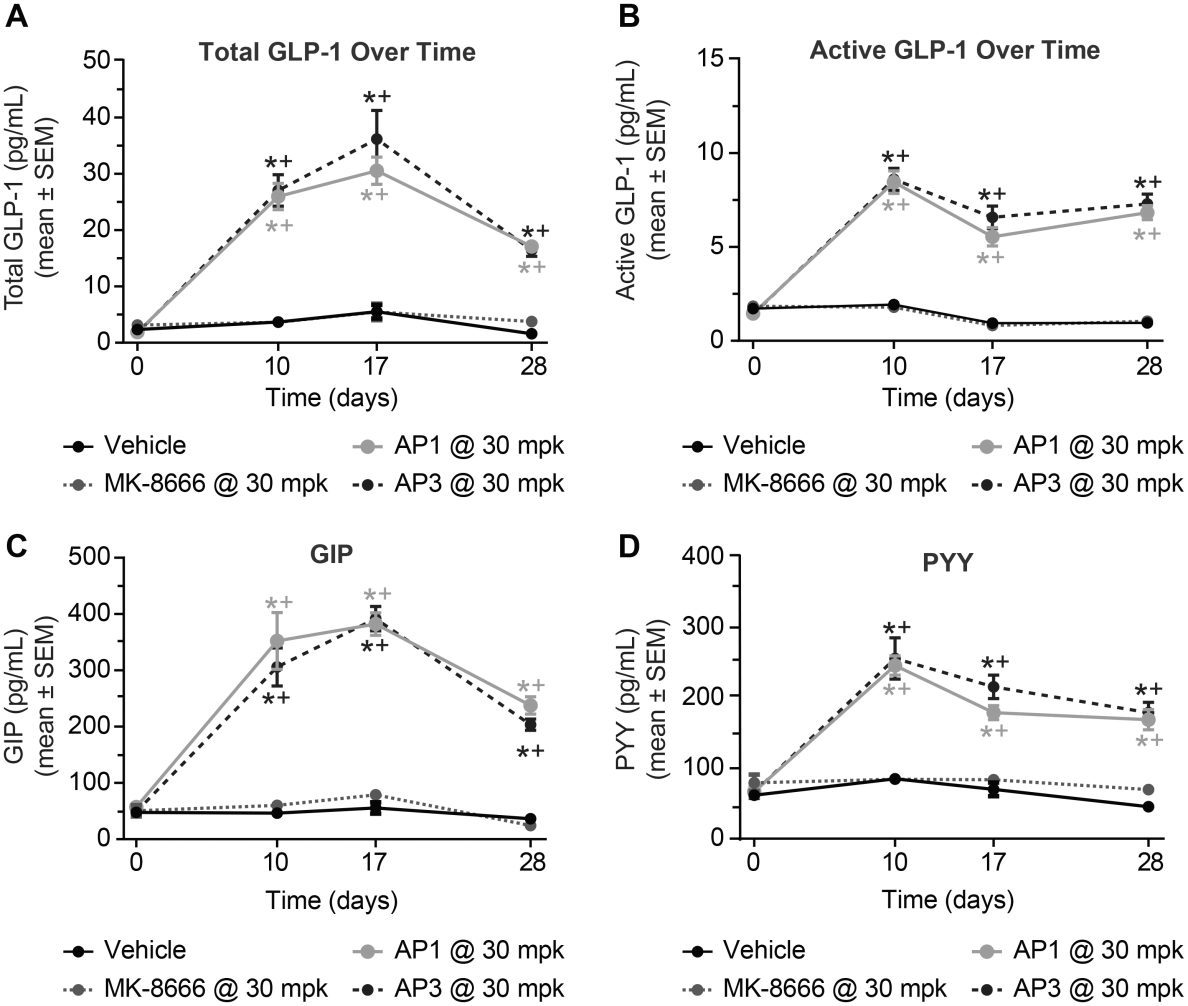
GK rat incretin hormones are affected by 28 days treatment with AgoPAMs or partial agonist. Both AgoPAMs significantly increased total (A) and active (B) GLP-1 throughout the 4 weeks of treatment. Other gut hormones, GIP (C) and PYY (D), were significantly elevated by the AgoPAM treatments. In contrast, MK-8666 had no effect on gut hormones. Data are mean ± SEM with analysis via ANOVA followed by Tukey’s posttest. * *P < 0*.*05* vs. Vehicle, ^+^ *P < 0*.*05* vs. MK-8666.

**Fig 5:**
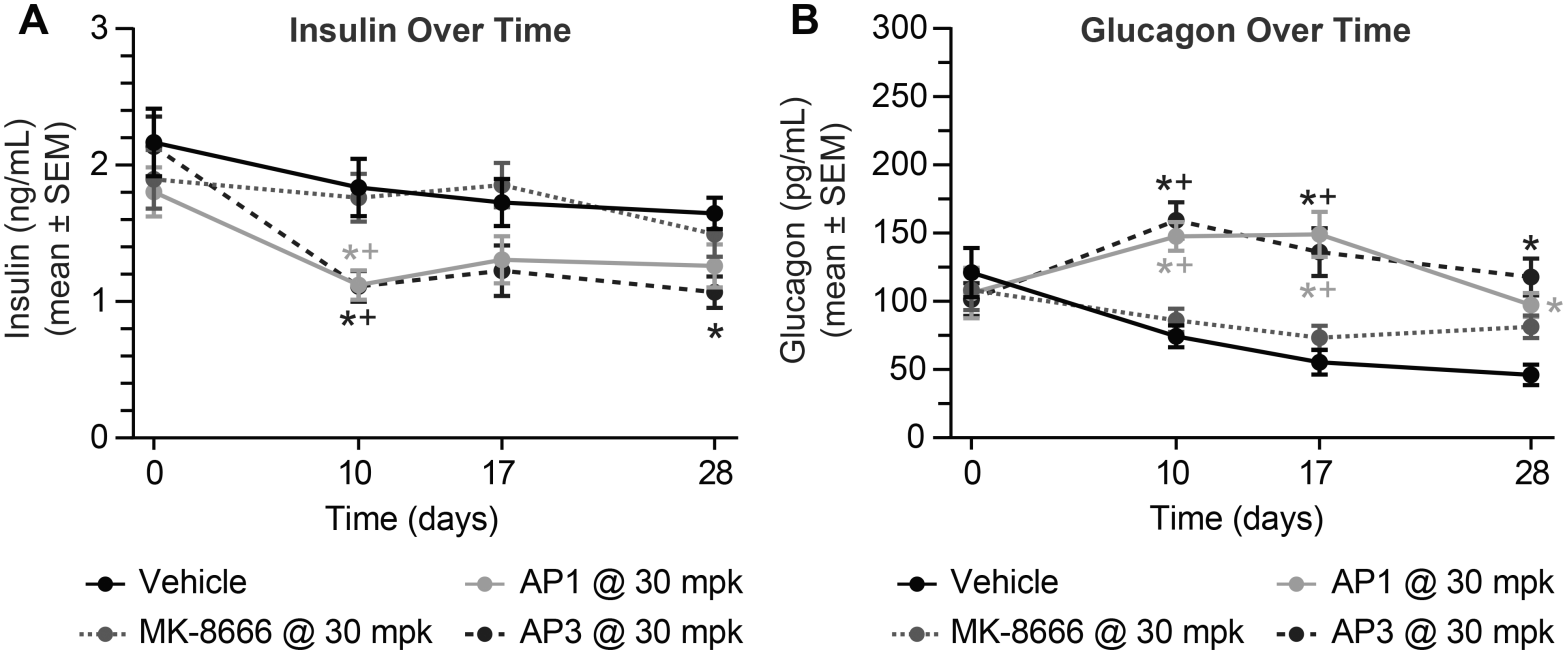
GK rat glucagon but not insulin affected by 28 days treatment with AgoPAMs or partial agonist. Little effect on insulin was seen in any treatment group (A). Treatment with AgoPAMs caused a significant and sustained increase in glucagon, while MK-8666 showed a trend of increasing glucagon at 28 days (B). Data are mean ± SEM with analysis via ANOVA followed by Tukey’s posttest. * *P < 0*.*05* vs. Vehicle; ^+^ *P < 0*.*05* vs. MK-8666.

More striking differentiation between the AgoPAMs and the partial agonist relates to the various pancreatic and gut hormones that are elevated with treatment. Both AgoPAMs significantly increased total and active GLP-1, GIP, and PYY throughout the 4 weeks of treatment (*P < 0*.*05*, Fig 4A–4D). In contrast, MK-8666, the partial agonist, had no effect on the gut hormones. The partial agonist also showed no effects on insulin chronically, while the AgoPAMs showed a slight drop in insulin (Fig 5A). Treatment with AgoPAMs caused a significant and sustained increase in glucagon, while MK-8666 only showed a trend of increasing glucagon on day 28 (Fig 5B).

### GPR40 AgoPAM effects on glucagon secretion require GPR40

**Fig 6:**
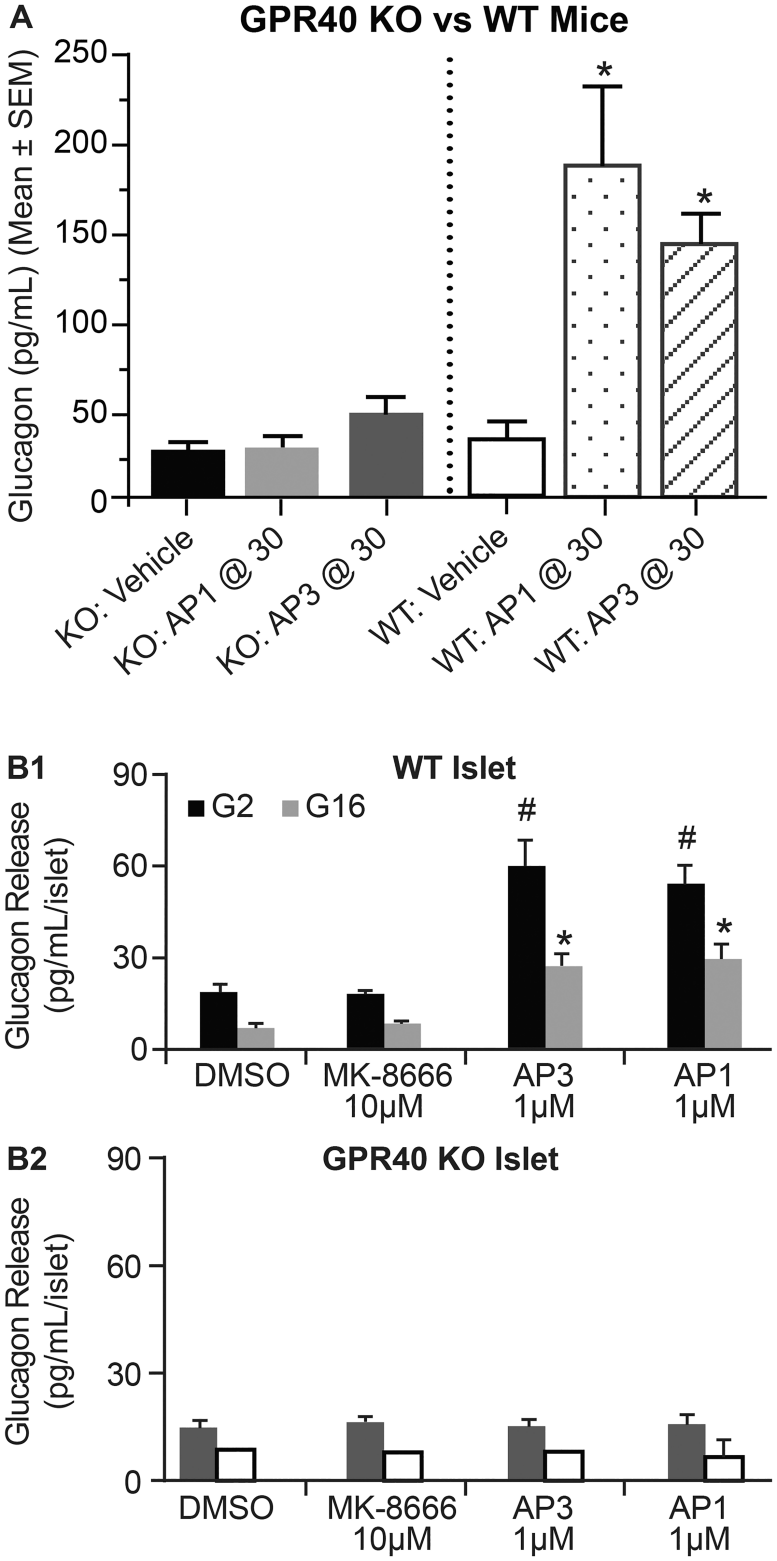
The effects of GPR40 ago-PAMs on glucagon release require GPR40. In vivo, treatment of mice with AgoPAMs AP1 or AP3 increase circulating glucagon in WT but not GPR40 -/- mice at 1h post dose. Ex-vivo, treatment of islets with GPR40 AgoPAMs, AP1 and AP3, but not MK-8666 significantly elevated glucagon secretion from WT islets (B1). Such sustained effect was abolished in GPR40^-/-^ islets (B2). Data are mean ± SEM with analysis via ANOVA followed by Dunnett’s posttest. * *P*, *#P < 0*.*05* vs. Vehicle.

To determine whether the glucagon effects seen with the AgoPAMs were target mediated, wild type and GPR40-/- mice were treated acutely with vehicle, AP1 at 30 mg/kg, or AP3 at 30 mg/kg. The wild type mice showed a significant increase in glucagon with AgoPAM treatment, while the GPR40-/- mice did not (P < 0.05, Fig 6A). This suggests the glucagon response to AgoPAM treatment requires the GPR40 receptor and is considered a target mediated effect. To understand whether changes in insulin or glucose levels play a role in the AgoPAM-mediated changes in glucagon, the effects of AgoPAMs vs. partial agonists on insulin and glucagon secretion were measured in isolated islets from wild type and GPR40^-/-^ mice. AgoPAMs significantly increased glucagon secretion from wild type mouse islets under both basal and high glucose levels but had no such effects on islets from the GPR40^-/-^ mice (P< 0.05, Fig 6B). In a similar paradigm, partial agonists exhibited no effect on glucagon under basal or high glucose levels ex vivo. Together these data demonstrate that unlike partial agonists, GPR40 AgoPAMs stimulate glucagon secretion from islets in a glucose independent fashion and require GPR40 to elicit this effect.

### GPR40 AgoPAM increases insulin secretion directly and through increases in GLP-1

**Fig 7:**
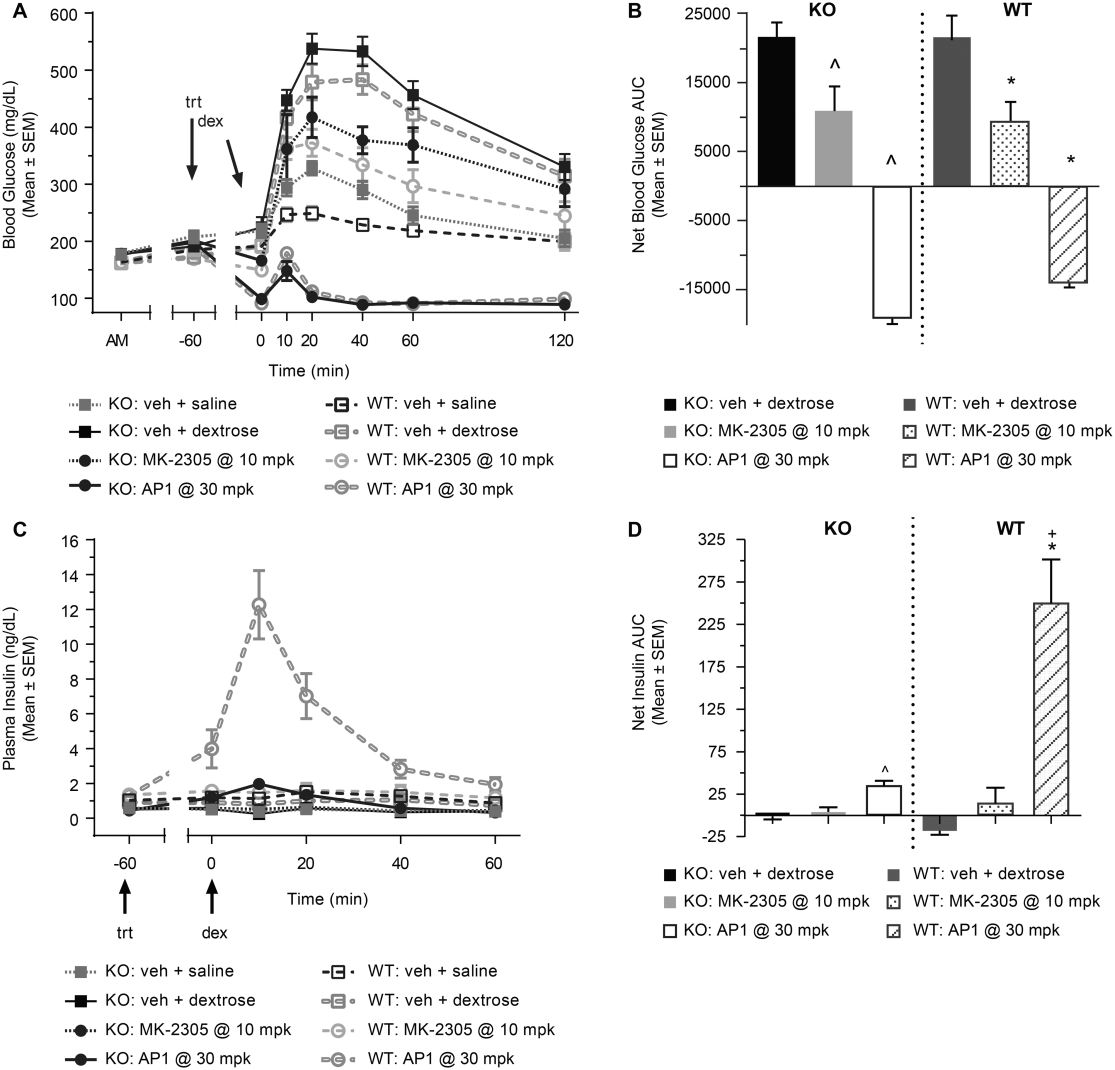
GLP-1R^-/-^ mice vs. wild type mice during an IPGTT. After treatment with MK-2305 at 10 mg/kg or AP1 at 30 mg/kg and a dextrose challenge, blood glucose excursion was reduced to similar levels in both the knockout and wild type mice compared to the respective vehicle groups (A, B). AP1 administered at a maximally efficacious dose increased insulin following the dextrose challenge in both knockout and wild type mice, but a much greater effect was seen in the wild type mice (C, D). The partial agonist MK-2305 administered at a maximally efficacious dose showed no effect in insulin for either the knockout or wild type mice. Data are mean ± SEM with analysis via ANOVA followed by Tukey’s posttest. ^ *P < 0*.*05* vs. GLP-1R^-/-^ Vehicle; * *P < 0*.*05* vs. WT Vehicle; ^+^ *P < 0*.*05* vs. GLP-1R^-/-^ AP1.

During an IPGTT with GLP-1R^-/-^ mice and wild type mice, treatment with the partial agonist MK-2305 and the AgoPAM reduced blood glucose excursion to a similar extent in both types of mice compared to the respective vehicle groups (Fig 7A and 7B). The AgoPAM showed a much greater reduction in glucose than the partial agonist. The similarity in responses of the GLP-1R^-/-^ mice and the wild type mice suggests that GLP-1 secretion is not required for the enhanced glucose lowering seen with the AgoPAM treatment in this acute experimental setting. When insulin is co-measured with glucose, the partial agonist showed no apparent changes in either the knockout or wild type mice (Fig 6C and 6D). The AgoPAM induced a significant elevation of insulin in the GLP-1R-/-mice, but an even greater increase was seen in the wild type mice (P < 0.05). These data demonstrate that unlike the partial agonists, the glucose dependent effects of GPR40 AgoPAMs have both GLP-1 dependent and GLP-1 independent components to it, such that AgoPAMs effect on GLP-1 secretion may enhance their GSIS above that observed with the partial agonist.

### Effects of AgoPAMs vs. partial GPR40 agonists on glucose metabolism in vivo

**Fig 8:**
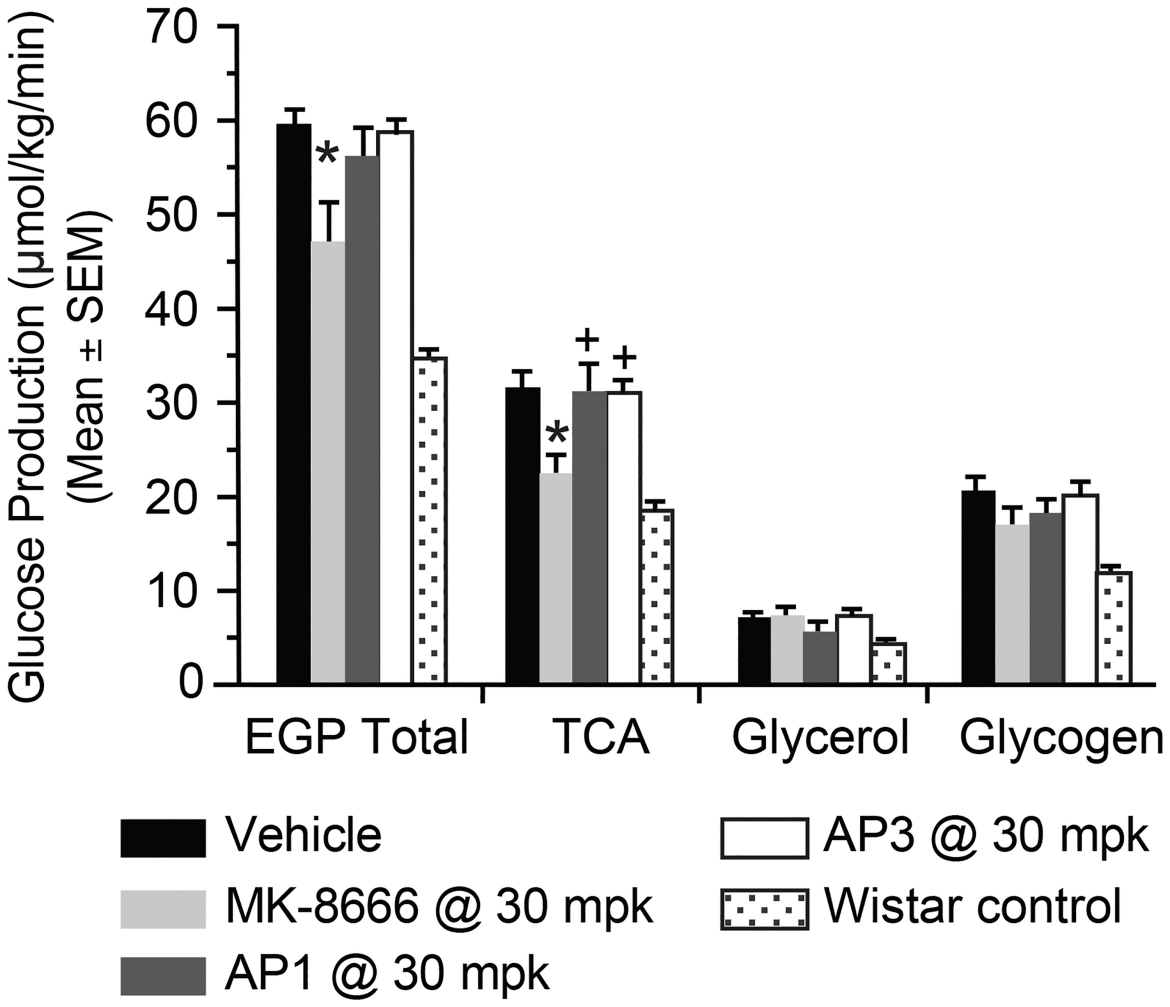
Total endogenous glucose production (EGP) and contributing fluxes in GK rats following chronic treatment with GPR40 partial agonist (MK-8666) or AgoPAMs (AP1 and AP3). Data are mean ± SEM with analysis via ANOVA followed by Tukey’s posttest. * *P < 0*.*05* vs. Vehicle; ^+^ *P < 0*.*05* vs. MK-8666.

**Fig 9:**
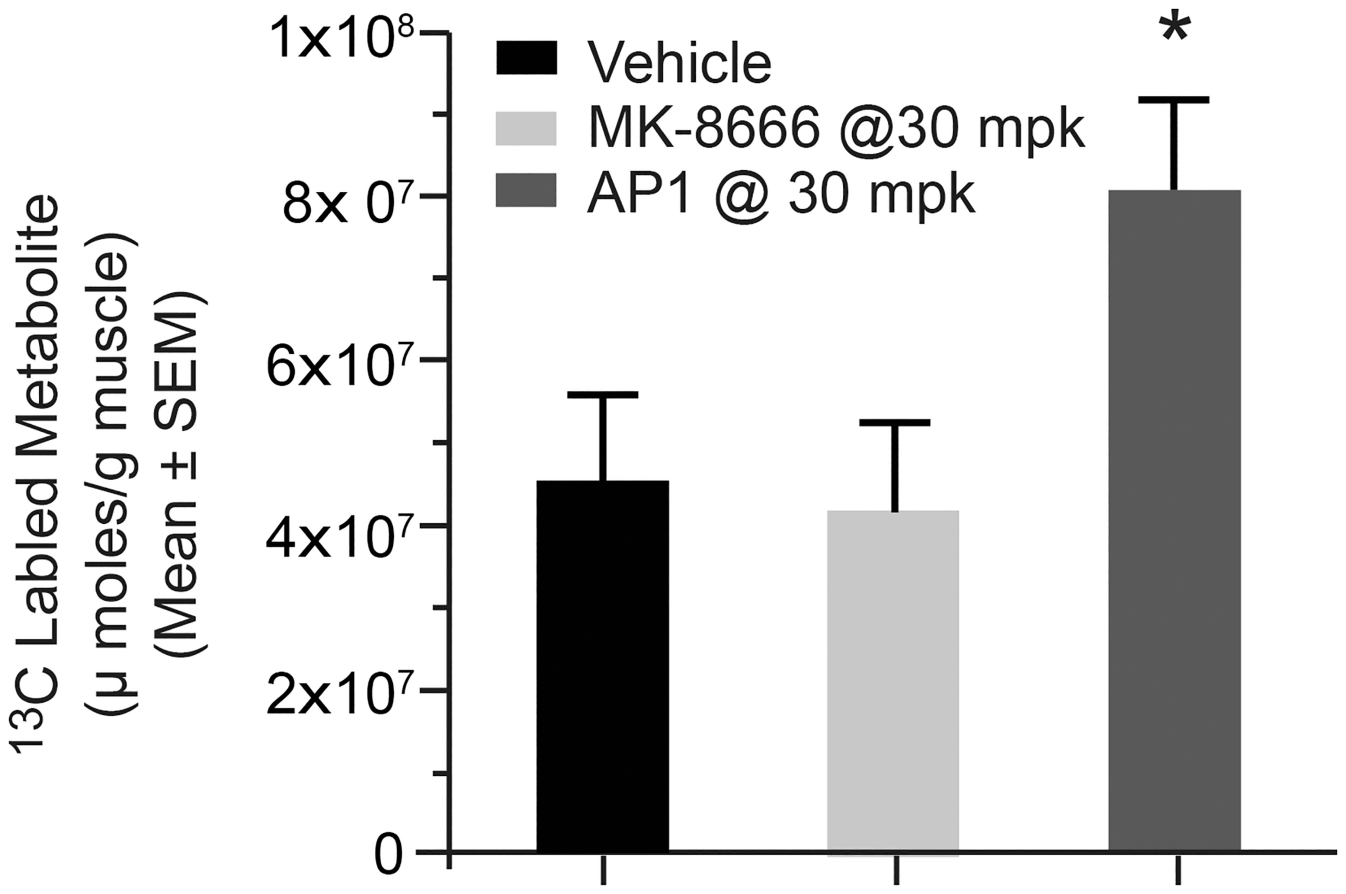
Skeletal muscle 13C-glycogen levels following a 13C-GTT in GK rats chronically treated with GPR40 partial agonist (MK-8666) or AgoPAM (AP1). Data are mean ± SEM with analysis via ANOVA followed by Tukey’s posttest. * *P < 0*.*05* vs. Vehicle; ^+^ *P < 0*.*05* vs. MK-8666.

To better understand how these hormonal effects may influence glucose metabolism and further explore the differential effects of partial agonists and AgoPAMs in vivo, we employed two complimentary tracer techniques to compare and contrast partial agonist and AgoPAM effects (Figs 8 and 9). Following chronic treatment, the GPR40 partial agonist MK-8666 reduced EGP; a reduction primarily mediated via decreases in gluconeogenesis from TCA cycle substrates (Fig 8). Interestingly, neither of the AgoPAM compounds had a significant effect on EGP, suggesting a different mechanism of action for this class of GPR40 agonists. To further investigate this potential differential mechanism, we employed a 13C-GTT following sub-chronic treatment with GPR40 partial agonist and AgoPAM compounds and measured accumulation of 13C-glycogen in skeletal muscle (Fig 9). Samples from rats treated with AP1 had increased 13C-glycogen levels compared to those treated with MK-8666 or vehicle, suggesting that AP1 stimulates increased muscle glucose uptake and storage as glycogen during a GTT. Taken together, the findings from these tracer studies suggest a difference in mechanisms of action for glucose lowering with GPR40 partial agonist vs. AgoPAM compounds.

## Discussion

These data highlight the differences between GPR40 partial agonists and AgoPAMs on key hormones involved in glucose homeostasis in vivo. AgoPAMs had first been described as binding to different sites on the receptor and eliciting a higher level of receptor activation compared to partial agonists in vivo [1]. In addition, GPR40 AgoPAMs are known to work coordinately with long chain free fatty acids to modulate dose response curves in vitro whereas partial agonists do not [4]. These molecular features are likely contributing to some of the in vivo differences observed when comparing the acute effects of partial agonists and AgoPAMs in human islets in vitro and mice in vivo, where AgoPAMs drive higher responses for both insulin and GLP-1 secretion compared to partial agonists [22]. Further, these hormonal effects are observed alongside greater glucose lowering suggesting a possible causal link between these two observations. The data we described in this manuscript and others [5,23] extend these initial reports of greater efficacy with GPR40 AgoPAMs beyond what was first observed acutely in mice and cells to the chronic setting in the diabetic GK rat. Furthermore, these studies used two structurally distinct AgoPAM compounds dosed at levels aimed to achieve comparable percent target engagement as the partial GPR40 agonists used. All compounds were dosed at maximally efficacious doses with regards glucose lowering with in their respective compound class (partial agonist or Ago PAM). Here, glucose was lowered as early as 2h post treatment and the glucose lowering efficacy with both partial agonists and AgoPAMs was maintained for up to 28 days of dosing. These data are in agreement with our previous study reported by Plummer et al [5]. However, these data also further differentiate partial vs. AgoPAM effects on glucose lowering where non-fasted blood glucose is decreased significantly more so with AgoPAMs compared to partial agonists. These data suggest the potential for further improvement on the glycemic profile. These are the first data suggesting such differentiation in the post-prandial setting beyond what can be shown in a glucose tolerance test.

Chronic treatment with AgoPAMs appears to affect food intake and body weight as well. Both AgoPAMs tested reduce these parameters compared to partial agonists that are otherwise body weight neutral. Reductions of food intake and body weight may contribute in part to the chronic changes in glucose that we observed. Further examination of the mechanisms involved in the effects of the AgoPAM vs. partial agonists on body weight and food intake are described in a separate manuscript with models and experimental conditions more conventionally used to characterize these effects [23]. Because the GK rat is a lean insulin deficient rodent model of type 2 diabetes, we focused most of our studies on the pharmacological differences between GPR40 partial agonists and AgoPAMs on the hormonal and metabolic regulation of glucose homeostasis.

GLP-1, GIP, and PYY were all elevated chronically with AgoPAM but not with GPR40 partial agonist treatment in the GK rat. Though previous studies had demonstrated GLP-1 and GIP were increased acutely, the present study surpasses the original observations at 60 min in mice [1] with observations of persistent elevations in the GK rat that extend out to 28 days and add observations of PYY to the incretin profile. These data provide no evidence for tachyphylaxsis or loss of effects in vivo and demonstrate impressive levels of incretin secretions compared to GPR40 partial agonist treatment where there is little to no effect on these incretins compared to baseline. It is intriguing to hypothesize that the levels of incretin secretion achieved with AgoPAM treatment could contribute to increases in glucose dependent insulin secretion and the post prandial improvements in glucose control. To address this question we examined the effect of GPR40 partial agonists vs. AgoPAMs in the GLP-1R KO mouse during a GTT with insulin-co-measurement. Indeed this experiment suggests there are contributions of both the intrinsic GPR40 receptor mediated properties as well as a GLP-1 mediated component of the enhanced insulin secretion of an AgoPAM vs. a partial agonist. Unlike the studies conducted by Liu acutely in the mouse setting, designing studies of this nature in the chronic rat setting are quite complicated and cumbersome due to 1) the lack of GLP-1R KO rats, and 2) the poor selectivity and PK properties of antagonists available to pharmacologically block the GLP-1 receptor in a chronic fashion. Thus our mouse data suggesting GPR40 AgoPAMs increase glucose dependent insulin secretion both directly and indirectly to improve glucose beyond that of a GPR40 partial agonist can only be inferred upon the GK rat, and if the mechanism translates, to the human.

Given the increases in incretins like GLP-1 are known to influence insulin and glucagon, two key hormones regulating glucose homeostasis; we compared the effects of GPR40 partial agonists and AgoPAMs on these hormones with surprising results. As expected, insulin was elevated acutely with both AgoPAM and partial agonist treatment in a glucose dependent manner. The effects of AgoPAMs on the changes in insulin were not different from partial agonists in that neither demonstrated major changes during fasting with chronic treatment. AgoPAM treatment resulted in a decrease in insulin levels but this change was not statistically significant at all time points and it is unclear whether the effect is physiologically meaningful. These results were anticipated as glucose was normalized under these conditions, so insulin secretion was not enabled. Changes in the prandial setting were likely, given small changes can be observed in insulin secretion after chronic treatment during a GTT. However, monitoring insulin changes over multiple time points (such as during feeding) in the chronic setting was not feasible in the available experimental paradigms.

Interestingly, we observed an acute increase in glucagon levels as early as 1h after the first dose of an AgoPAM. Though small changes were also observed with the partial agonist, they were not statistically significant and were not observed past the first day of dosing. In contrast the elevation in glucagon with AgoPAM treatment was consistent throughout the 28d of treatment. To assure the effects of AgoPAMs on glucagon were in fact on target (i.e. GPR40- mediated), we show the elevations in glucagon in wild type mice are not observed in the GPR40 KO mouse. These results are exceptionally surprising in that the increase in glucagon occurred in conjunction with a significant increase in circulating GLP-1. GLP-1 is known to increase insulin secretion and satiety, and to decrease glucagon [24]. While we show there are effects of AgoPAMs on insulin secretion (this manuscript) and satiety [23] that are likely mediated in part by GLP-1, glucagon is elevated together with GLP-1. This paradoxical pharmacological effect is the first report of an AgoPAM acting to elevate these two hormones in this fashion in both mice and rats in vivo. The persistence of this effect in the chronic setting is also unusual, and implications of this phenomenon are the subject of ongoing research.

To further probe the effects of AgoPAMs on glucagon secretion, we compared the effects of GPR40 partial and AgoPAM agonists on islet hormone secretions ex-vivo. In vitro investigations of the effects of partial vs. AgoPAM effects on islet secretions revealed GPR40 AgoPAMs increase glucagon secretion directly from islets in a seemingly glucose and insulin independent fashion. AgoPAMs increased glucagon levels in cultures of islets at basal and high glucose in both the presence and absence of concomitant increases in insulin secretion in islet cultures. Similar to effects on insulin, AgoPAMs increase glucagon secretion in a GPR40 dependent fashion as the event was not observed in islets cultured from GPR40 KO mice. This hormonal phenomenon is unusual given glucagon is regulated primarily by high glucose levels whereby glucagon secretion by the alpha cell is suppressed [25]. Here we show that AgoPAMs can increase glucagon secretion under conditions of high glucose both in vivo and in islet cultures. We also observe this AgoPAM mediated increase take place in the presence of elevated GLP-1 in vivo. This too is unexpected as elevations in GLP-1 have also been shown to decrease glucagon [26]. Together these data provide new insight into glucagon regulation by GPR40 AgoPAMs, and it is impressive how AgoPAM treatment can maintain high levels of glucagon secretion under conditions that normally do not support elevations of this hormone (high GLP-1 and glucose). Future studies to examine the effects of chronic AgoPAM treatment on islet function and viability would provide important insight to the potential for GPR40 AgoPAMs to become a therapeutic for type 2 diabetic patients where islet structure and function is altered as a part of the etiology of the disease.

Given the chronic elevations in both GLP-1 and glucagon with AgoPAM treatment, it is interesting to speculate that GPR40 AgoPAMs may provide similar metabolic benefits to those observed with GLP-1/glucagon co-agonist peptide therapeutics. These peptides have been under development for the treatment of type 2 diabetes for several years by large pharmaceutical companies. The preclinical data describing GPR40 AgoPAM small molecules and GLP-1/glucagon peptides show similarities in that they both demonstrate significant beneficial effects on glucose and bodyweight [27,28]. Perhaps future studies designed to compare and contrast these two potential anti-diabetic therapies may reveal more about the mechanisms mediating their beneficial effects, enable optimization, and aid in evaluating which of the two therapies may be more desirable for patients.

Our data suggest that GPR40 AgoPAM and partial agonists differ in the mechanisms that drive the differential glycemic profiles observed. Specifically, we show data suggesting that unlike partial agonists, AgoPAMs may lower glucose to a greater extent in the fed or post prandial state. Exploration of this observation with tracer methodologies revealed that the mechanisms mediating the difference in reductions in glucose during a GTT with AgoPAMs is due in part to enhanced glucose uptake and storage as skeletal muscle glycogen. We hypothesize that this difference in skeletal muscle glucose uptake may also contribute to the greater glucose lowering observed with AgoPAMs compared to partial GPR40 agonists in the fed state. More importantly, this finding provides mechanistic support for greater glucose control and potentially improved glycemic profiles with AgoPAMs compared to partial agonist treatment. Interestingly, only the partial agonist lowered endogenous glucose production whereas the AgoPAM did not. Though this was surprising given that both reduce glucose in the fasted state, we hypothesize that the elevations in glucagon observed with AgoPAM treatment may contribute to this difference in glucose metabolism. We also hypothesize that the changes in fasted glucose with AgoPAM treatment may also be in part through increases in glucose uptake; however, specific studies designed to assess this feature are needed to explore this aspect of the AgoPAM mechanism of action.

Further studies are needed to determine whether all these hormonal and metabolic effects translate to higher species and, more importantly, to human subjects. Overall, these data highlight the value of engaging the full potential of the GPR40 receptor. We have confirmed and greatly extended the initial acute observations in mice with AgoPAM treatment on glucose and GLP-1 to demonstrate chronic glucose lowering in the diabetic GK rat coupled with significant increases in GLP-1, GIP, and PYY. Interestingly we show that similar to partial agonists, AgoPAM treatment leads to transient increases in insulin observed when glucose is elevated. However, unlike partial agonists, AgoPAM amplification of glucose dependent insulin secretion is observed alongside chronic increases in circulating glucagon. The differences in effects observed between partial agonists and AgoPAMs are intriguing. Further evaluation of the AgoPAM effects on islet health and in higher species are warranted in hopes of continuing the development of this exciting therapeutic, where additional glycemic benefits and potential for benefits beyond glycaemia may be available for patients.

## Supporting information

S1 Fig Percent target engagement over 24h treatment based on pharmacokinetic sampling in the GK rat after a single dose of TAK-875, AP1, and AP3. (TIF)

S1 Table Percent target engagement over 24h treatment based on pharmacokinetic sampling in the GK rat after a single dose of TAK-875, AP1, and AP3. (TIF)

S2 Table Mean (SD) blood concentrations (uM) of GPR40 Agonists during chronic administration to GK rats. (TIF)

S3 Table Mean (SD) calculated GPR40 target engagement during chronic administration to GK rats. (TIF)

S1 File Data for Fig 1: GPR40 in vitro pharmacology across species. (XLSX)

S2 File Data for Fig 2A glucose over time (mg/dl). (XLS)

S3 File Data for Fig 2B plasma insulin over time (ng/ml). (XLS)

S4 File Data for Fig 2C plasma glucagon over time (pg/ml). (XLS)

S5 File Data for Fig 3A plasma glucose over time (mg/dl). (XLS)

S6 File Data for Fig 3B fed and fasted glucose on day 28. (XLS)

S7 File Data for Fig 3C food intake over time. (XLS)

S8 File Data for Fig 3D total body weight change over time. (XLS)

S9 File Data for Fig 4A total GLP-1 levels over time. (XLS)

S10 File Data for Fig 4B active GLP-1 levels over time. (XLS)

S11 File Data for Fig 4C GIP levels over time. (XLS)

S12 File Data for Fig 4D PYY levels over time. (XLS)

S13 File Data for Fig 5A plasma insulin over time (ng/ml). (XLS)

S14 File Data for Fig 5B plasma glucagon over time (pg/ml). (XLS)

S15 File Data for Fig 6A plasma glucagon over time (pg/ml). (XLS)

S16 File Data for Fig 6B1 and 6B2 islet glucagon (pg/ml/ islet). (XLS)

S17 File Data for Fig 7A blood glucose over time (mg/dl). (XLS)

S18 File Data for Fig 7B blood glucose Net AUC. (XLS)

S19 File Data for Fig 7C plasma insulin over time (ng/ml). (XLS)

S20 File Data for Fig 7D plasma insulin Net AUC. (XLS)

S21 File Data for Fig 8 glucose prodiction (umol/ kg/min). (XLS)

S22 File Data for Fig 9 C13 labled metabolite (umoles/g muscle). (XLS)

## References

[1] Luo J, Swaminath G, Brown SP, Zhang J, Guo Q, Chen M, et al A potent class of GPR40 full agonists engages the enteroinsular axis to promote glucose control in rodents. PloS One. 2012;7: e46300 doi: 10.1371/journal.pone.0046300 23056280 10.1371/journal.pone.0046300 PMC3467217

[2] Hauge M, Vestmar MA, Husted AS, Ekberg JP, Wright MJ, Di Salvo J, et al GPR40 (FFAR1)—Combined Gs and Gq signaling in vitro is associated with robust incretin secretagogue action ex vivo and in vivo. Mol Metab. 2015;4: 3–14. doi: 10.1016/j.molmet.2014.10.002 25685685 10.1016/j.molmet.2014.10.002 PMC4314522

[3] Yabuki C, Komatsu H, Tsujihata Y, Maeda R, Ito R, Matsuda-Nagasumi K, et al A novel antidiabetic drug, fasiglifam/TAK-875, acts as an ago-allosteric modulator of FFAR1. PloS One. 2013;8: e76280 doi: 10.1371/journal.pone.0076280 24130766 10.1371/journal.pone.0076280 PMC3794927

[4] Xiong Y, Swaminath G, Cao Q, Yang L, Guo Q, Salomonis H, et al Activation of FFA1 mediates GLP-1 secretion in mice. Evidence for allosterism at FFA1. Mol Cell Endocrinol. 2013;369: 119–129. doi: 10.1016/j.mce.2013.01.009 23403053 10.1016/j.mce.2013.01.009

[5] Plummer CW, Clements MJ, Chen H, Rajagopalan M, Josien H, Hagmann WK, et al Design and Synthesis of Novel, Selective GPR40 AgoPAMs. ACS Med Chem Lett. 2017; doi: 10.1021/acsmedchemlett.6b00443 28197316 10.1021/acsmedchemlett.6b00443 PMC5304298

[6] Burant CF. Activation of GPR40 as a therapeutic target for the treatment of type 2 diabetes. Diabetes Care. 2013;36 Suppl 2: S175–179. doi: 10.2337/dcS13-2037 23882043 10.2337/dcS13-2037 PMC3920793

[7] Briscoe CP, Peat AJ, McKeown SC, Corbett DF, Goetz AS, Littleton TR, et al Pharmacological regulation of insulin secretion in MIN6 cells through the fatty acid receptor GPR40: identification of agonist and antagonist small molecules. Br J Pharmacol. 2006;148: 619–628. doi: 10.1038/sj.bjp.0706770 16702987 10.1038/sj.bjp.0706770 PMC1751878

[8] Itoh Y, Hinuma S. GPR40, a free fatty acid receptor on pancreatic beta cells, regulates insulin secretion. Hepatol Res Off J Jpn Soc Hepatol. 2005;33: 171–173. doi: 10.1016/j.hepres.2005.09.028 16214394 10.1016/j.hepres.2005.09.028

[9] Drucker DJ, Philippe J, Mojsov S, Chick WL, Habener JF. Glucagon-like peptide I stimulates insulin gene expression and increases cyclic AMP levels in a rat islet cell line. Proc Natl Acad Sci. 1987;84: 3434–3438. 3033647 10.1073/pnas.84.10.3434 PMC304885

[10] Lan H, Hoos LM, Liu L, Tetzloff G, Hu W, Abbondanzo SJ, et al Lack of FFAR1/GPR40 does not protect mice from high-fat diet-induced metabolic disease. Diabetes. 2008;57: 2999–3006. doi: 10.2337/db08-0596 18678612 10.2337/db08-0596 PMC2570396

[11] Negoro N, Sasaki S, Mikami S, Ito M, Suzuki M, Tsujihata Y, et al Discovery of TAK-875: A Potent, Selective, and Orally Bioavailable GPR40 Agonist. ACS Med Chem Lett. 2010;1: 290–294. doi: 10.1021/ml1000855 24900210 10.1021/ml1000855 PMC4007909

[12] Brockunier LL, Chen H, Chobanian HR, Clements MJ, CRESPO A, Demong DE, et al. Antidiabetic bicyclic compounds [Internet]. WO2014130608 A1, 2014. http://www.google.com/patents/WO2014130608A1

[13] Hyde AM, Liu Z, Kosjek B, Tan L, Klapars A, Ashley ER, et al Synthesis of the GPR40 Partial Agonist MK-8666 through a Kinetically Controlled Dynamic Enzymatic Ketone Reduction. Org Lett. 2016;18: 5888–5891. doi: 10.1021/acs.orglett.6b02910 27802043 10.1021/acs.orglett.6b02910

[14] Lacy PE, Kostianovsky M. Method for the isolation of intact islets of Langerhans from the rat pancreas. Diabetes. 1967;16: 35–39. 5333500 10.2337/diab.16.1.35

[15] Li XN, Herrington J, Petrov A, Ge L, Eiermann G, Xiong Y, et al The role of voltage-gated potassium channels Kv2.1 and Kv2.2 in the regulation of insulin and somatostatin release from pancreatic islets. J Pharmacol Exp Ther. 2013;344: 407–416. doi: 10.1124/jpet.112.199083 23161216 10.1124/jpet.112.199083

[16] Tan CP, Feng Y, Zhou Y-P, Eiermann GJ, Petrov A, Zhou C, et al Selective small-molecule agonists of G protein-coupled receptor 40 promote glucose-dependent insulin secretion and reduce blood glucose in mice. Diabetes. 2008;57: 2211–2219. doi: 10.2337/db08-0130 18477808 10.2337/db08-0130 PMC2494688

[17] Wang S-P, Zhou D, Yao Z, Satapati S, Chen Y, Daurio NA, et al Quantifying rates of glucose production in vivo following an intraperitoneal tracer bolus. Am J Physiol Endocrinol Metab. 2016; doi: 10.1152/ajpendo.00182.2016 27651111 10.1152/ajpendo.00182.2016

[18] Jin ES, Jones JG, Merritt M, Burgess SC, Malloy CR, Sherry AD. Glucose production, gluconeogenesis, and hepatic tricarboxylic acid cycle fluxes measured by nuclear magnetic resonance analysis of a single glucose derivative. Anal Biochem. 2004;327: 149–155. doi: 10.1016/j.ab.2003.12.036 15051530 10.1016/j.ab.2003.12.036

[19] Myers RW, Guan H-P, Ehrhart J, Petrov A, Prahalada S, Tozzo E, et al Systemic pan-AMPK activator MK-8722 improves glucose homeostasis but induces cardiac hypertrophy. Science. 2017; eaah5582. doi: 10.1126/science.aah5582 28705990 10.1126/science.aah5582

[20] Lee AYH, Chappell DL, Bak MJ, Judo M, Liang L, Churakova T, et al Multiplexed Quantification of Proglucagon-Derived Peptides by Immunoaffinity Enrichment and Tandem Mass Spectrometry after a Meal Tolerance Test. Clin Chem. 2016;62: 227–235. doi: 10.1373/clinchem.2015.244251 26430077 10.1373/clinchem.2015.244251

[21] Lin DC-H, Guo Q, Luo J, Zhang J, Nguyen K, Chen M, et al Identification and pharmacological characterization of multiple allosteric binding sites on the free fatty acid 1 receptor. Mol Pharmacol. 2012;82: 843–859. doi: 10.1124/mol.112.079640 22859723 10.1124/mol.112.079640 PMC3477236

[22] Luo J, Nguyen K, Chen M, Tran T, Hao J, Tian B, et al Evaluating insulin secretagogues in a humanized mouse model with functional human islets. Metabolism. 2013;62: 90–99. doi: 10.1016/j.metabol.2012.07.010 22982177 10.1016/j.metabol.2012.07.010

[23] Gorski JN, Pachanski MJ, Mane J, Plummer CW, Souza S, Thomas-Fowlkes BS, et al GPR40 reduces food intake and body weight through GLP-1. Am J Physiol—Endocrinol Metab. 2017; doi: 10.1152/ajpendo.00435.2016 28292762 10.1152/ajpendo.00435.2016

[24] Campbell JE, Drucker DJ. Pharmacology, physiology, and mechanisms of incretin hormone action. Cell Metab. 2013;17: 819–837. doi: 10.1016/j.cmet.2013.04.008 23684623 10.1016/j.cmet.2013.04.008

[25] Gerich JE, Charles MA, Grodsky GM. Characterization of the Effects of Arginine and Glucose on Glucagon and Insulin Release from the Perfused Rat Pancreas. J Clin Invest. 1974;54: 833–841. doi: 10.1172/JCI107823 4430717 10.1172/JCI107823 PMC301623

[26] Holst JJ, Christensen M, Lund A, de Heer J, Svendsen B, Kielgast U, et al Regulation of glucagon secretion by incretins. Diabetes Obes Metab. 2011;13 Suppl 1: 89–94. doi: 10.1111/j.1463-1326.2011.01452.x 21824261 10.1111/j.1463-1326.2011.01452.x

[27] Day JW, Gelfanov V, Smiley D, Carrington PE, Eiermann G, Chicchi G, et al Optimization of co-agonism at GLP-1 and glucagon receptors to safely maximize weight reduction in DIO-rodents. Biopolymers. 2012;98: 443–450. doi: 10.1002/bip.22072 23203689 10.1002/bip.22072

[28] Pocai A, Carrington PE, Adams JR, Wright M, Eiermann G, Zhu L, et al Glucagon-like peptide 1/glucagon receptor dual agonism reverses obesity in mice. Diabetes. 2009;58: 2258–2266. doi: 10.2337/db09-0278 19602537 10.2337/db09-0278 PMC2750209

