# Supplementary figures and images for: GPR40 partial agonists and AgoPAMs: Differentiating effects on glucose and hormonal secretions in the rodent
Source: PLoS One. 2017 Oct 20;12(10):e0186033. doi: 10.1371/journal.pone.0186033 (PMC5650142; doi:10.1371/journal.pone.0186033)

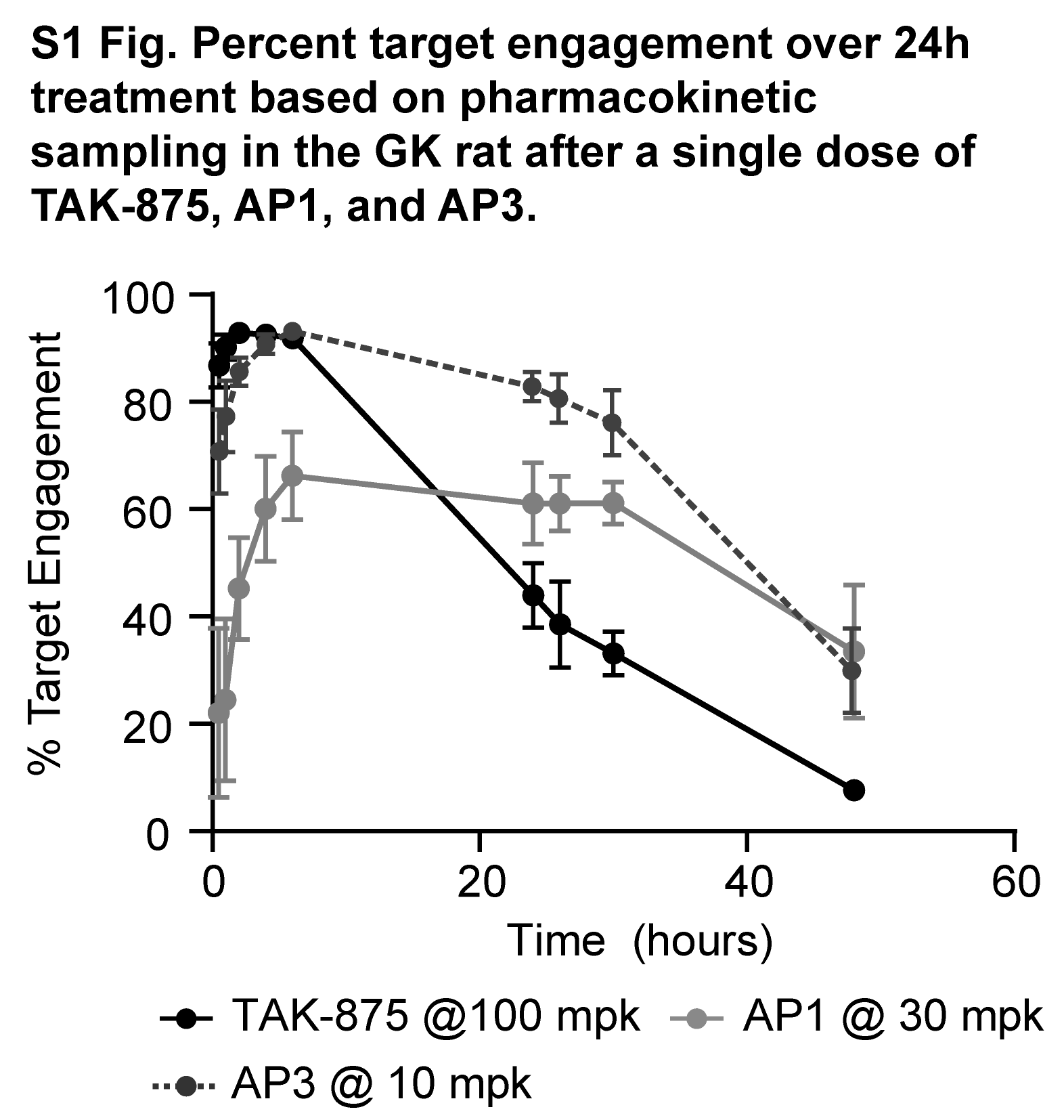

Supplement: S1 Fig — (TIF) [file pone.0186033.s001.tif]

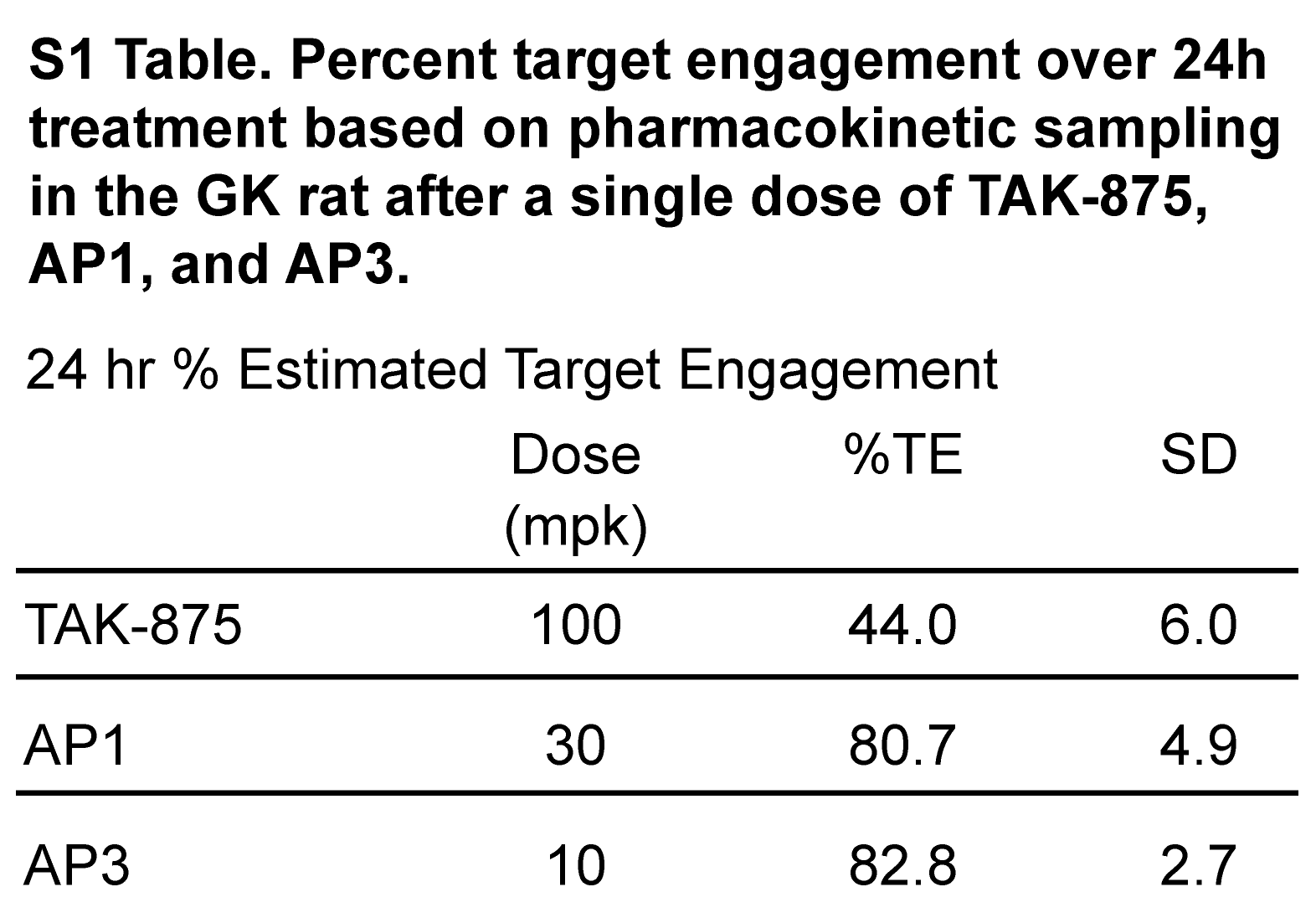

Supplement: S1 Table — (TIF) [file pone.0186033.s002.tif]

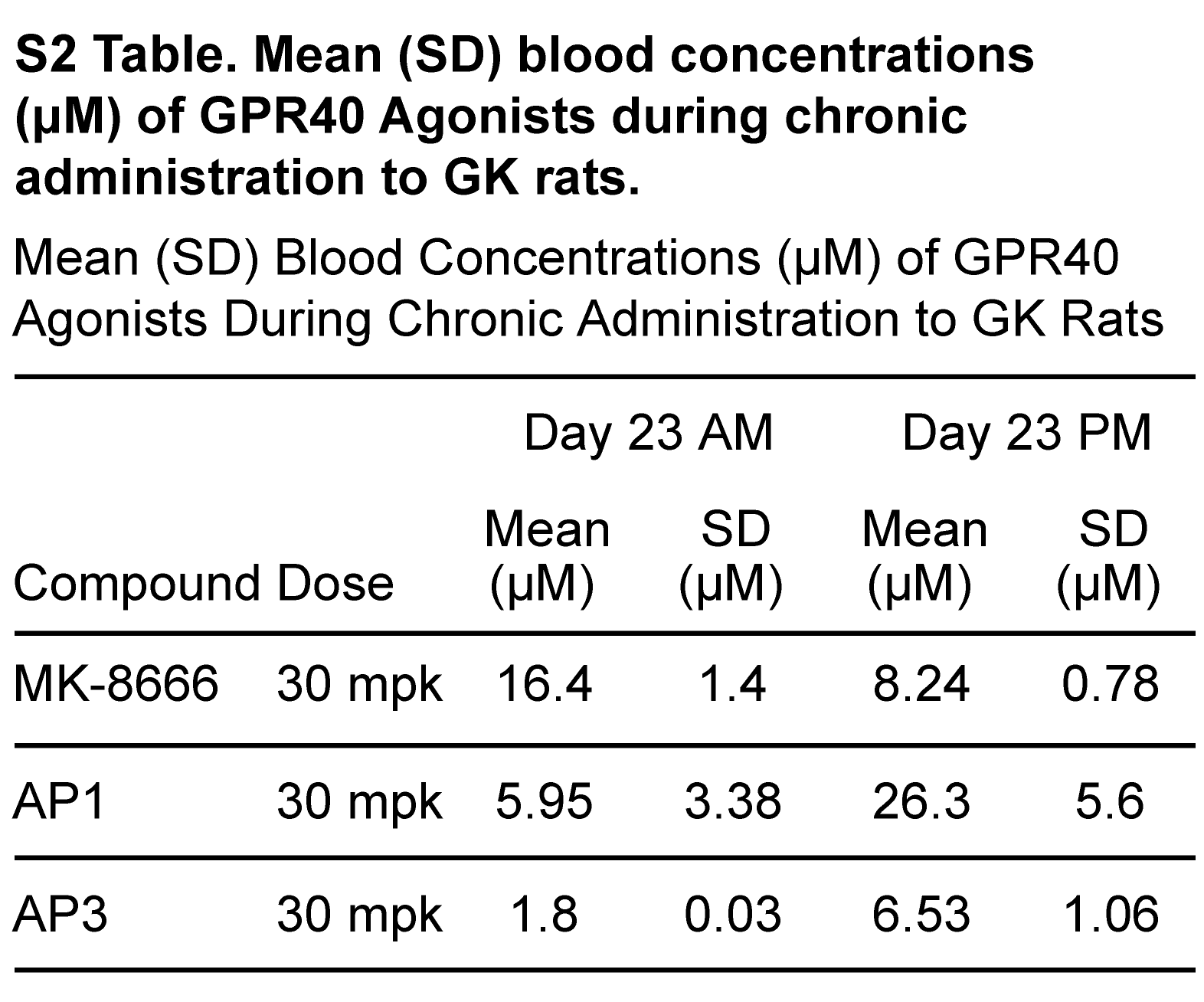

Supplement: S2 Table — (TIF) [file pone.0186033.s003.tif]

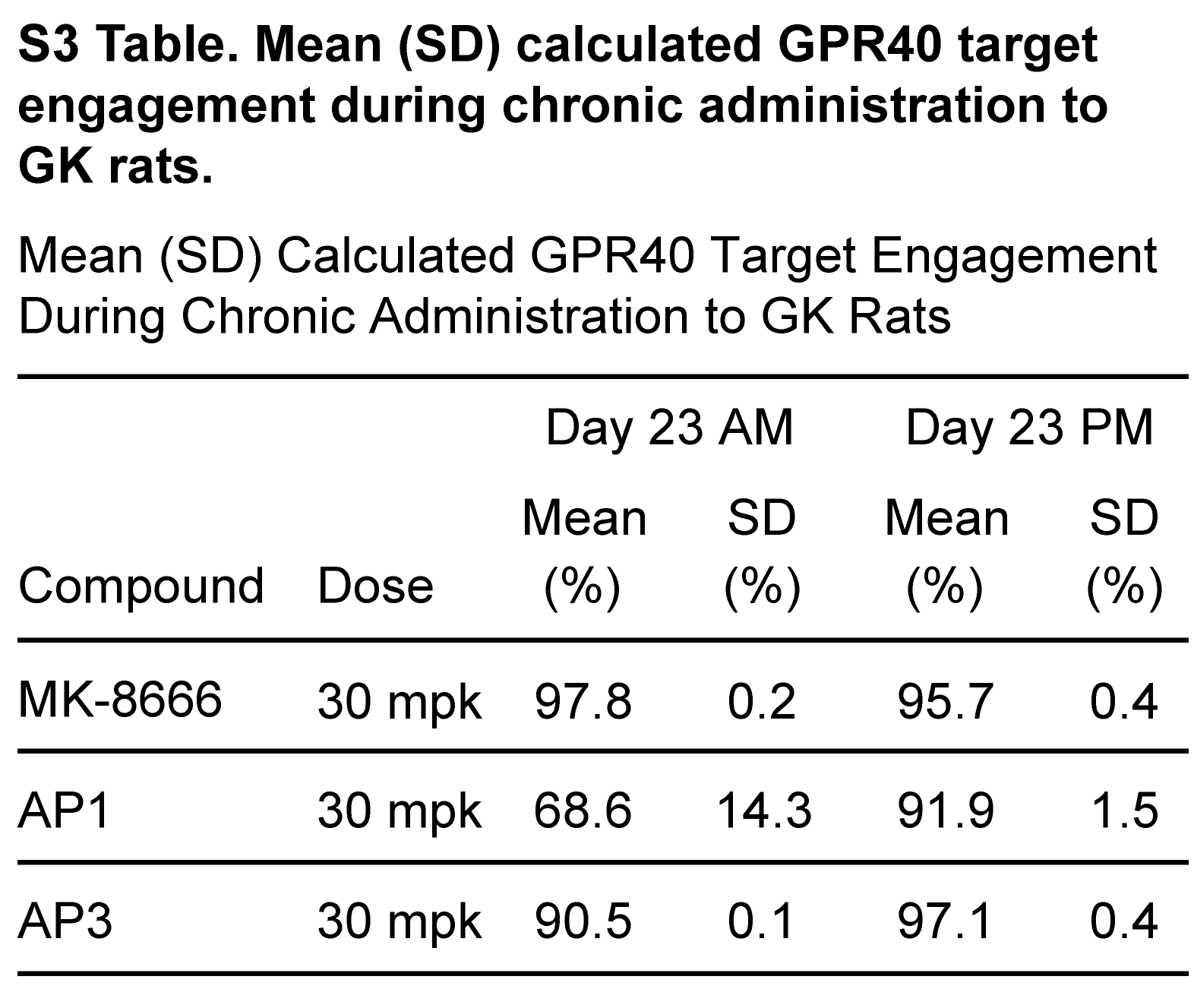

Supplement: S3 Table — (TIF) [file pone.0186033.s004.tif]
